# A Comprehensive Prognostic and Immune Analysis of SLC41A3 in Pan-Cancer

**DOI:** 10.3389/fonc.2020.586414

**Published:** 2021-01-14

**Authors:** Jun Liu, Shanqiang Zhang, Wenjie Dai, Chongwei Xie, Ji-Cheng Li

**Affiliations:** ^1^Medical Research Center, Yue Bei People’s Hospital, Shantou University Medical College, Shaoguan, China; ^2^Institute of Cell Biology, Zhejiang University, Hangzhou, China

**Keywords:** SLC41A3, pan-cancer, the Cancer Genome Atlas, immune analysis, nomogram

## Abstract

SLC41A3, as a member of the 41^st^ family of solute carriers, participates in the transport of magnesium. The role of SLC41A3 in cancer prognosis and immune regulation has rarely been reported. This study was designed to analyze the expression status and prognostic significance of SLC41A3 in pan-cancers. The mRNA expression profiles of SLC41A3 were obtained from The Cancer Genome Atlas (TCGA), the Genotype-Tissue Expression (GTEx), the Broad Institute Cancer Cell Line Encyclopedia (CCLE), and the International Cancer Genome Consortium (ICGC). The Cox regression and Kaplan-Meier analyses were used to evaluate the prognostic value of SLC41A3 in pan-cancer. Furthermore, the correlation between SLC41A3 expression and immune cells infiltration, immune checkpoint, mismatch repair (MMR), DNA methyltransferase (DNMT), tumor mutation burden (TMB), and microsatellite instability (MSI) were calculated using data form TCGA database. The results showed that the expression of SLC41A3 was down-regulated in kidney renal clear cell carcinoma (KIRC), and was associated with poor overall survival and tumor-specific mortality. Whereas, the expression of SLC41A3 was up-regulated in liver hepatocellular carcinoma (LIHC), and the results of Cox regression analysis revealed that SLC41A3 was an independent factor for LIHC prognosis. Meanwhile, a nomogram including SLC41A3 and stage was built and exhibited good predictive power for the overall survival of LIHC patients. Additionally, correlation analysis suggested a significant correlation between SLC41A3 and TMB, MSI, MMR, DNMT, and immune cells infiltration in various cancers. The overall survival and disease-specific survival analysis revealed that the combined SLC41A3 expression and immune cell score, TMB, and MSI were significantly associated with clinical outcomes in ACC, LIHC, and UVM patients. Therefore, we proposed that SLC41A3 may serve as a potential prognostic biomarker for cancer.

## Introduction

Worldwide, cancer has seriously jeopardized public health, and the incidence and mortality of cancer are rapidly increasing every year (1). Among the most common cancers, lung, pancreatic, and liver cancers are responsible for high mortality rate worldwide (1). Despite considerable effort has been made to enhance the diagnosis and treatment of cancer, the 5-year survival rate is still disheartening (2). Meanwhile, the financial burden of cancer has being heavily imposed on countries all over the world (3).Therefore, there is an urgent need to search for new methods to diagnose and treat cancer. Currently, the application of cancer biomarkers has drawn great attention of scientists, thereby promoting researchers to explore novel cancer biomarkers (4–6).

SLC41A3 is a member of the 41^st^ family of solute carriers, which can play a significant role in the magnesium (Mg^2+^) transport (7–9). SLC41A3 is a mitochondrial protein, and the over-expression of SLC41A3 could result in the efflux of Mg^2+^ from the mitochondria (10). It has been demonstrated that aberrant expression of SLC41A3 is associated with various diseases, including hypertension, Parkinson’s disease, and nephronophthisis (11, 12). However, the role of SLC41A3 in pan-cancer remains unclear.

In recent years, DNA methylation and the tumor microenvironment (TME) have earned substantial interest (13, 14). The role of DNA methylation biomarkers in the diagnosis and prognosis of different cancers has been described in previous studies, providing insight into the underlying mechanisms of tumor evolution (15–17). Also, TME has been recognized as a main contributor to cancer occurrence and progression (18, 19). The aggregation of various infiltrating immune cells in the TME has been found to be related to cancer development. These infiltrating immune cells, such as tumor-associated-macrophages, regulatory T cells, B cells, and natural killer cells do not inhibit the cancer cell growth, but can play a role in the immune escape of cancer (19–21). The inhibitory checkpoints, such as programmed cell death ligand-1 (PD-L1) and cytotoxic T lymphocyte associated antigen 4 (CTLA4) have been demonstrated to inhibit anti-tumor immunity, leading to evade the host immune attack (22, 23). However, to date, the outcome of cancer immunotherapy is far from optimal (19). Therefore, it is necessary to explore novel potential targets for cancer immunotherapy.

In this study, we examined the expression of SLC41A3 in normal tissues, various cell lines, and pan-cancer. Meanwhile, we also evaluated the prognostic value of SLC41A3 in pan-cancer based on The Cancer Genome Atlas (TCGA) dataset. Subsequently, we analyzed the association between the SLC41A3 expression levels and immune cells infiltration, immune checkpoint, mutation burden, and DNA methylation. The International Cancer Genome Consortium (ICGC) and the Gene Expression Omnibus (GEO) databases were used to verify the expression level and prognostic value of SLC41A3 in liver hepatocellular carcinoma (LIHC).

## Methods and Materials

### Data Collection and Processing

The gene expression data and RNA sequencing of TCGA pan-cancer, cancer cell line encyclopedia (CCLE), and the Genotype-Tissue Expression (GTEx) were extracted from the University of California Santa Cruz (UCSC) Xena browser (https://xena.ucsc.edu/), (https://portals.broadinstitute.org/ccle/data), and (https://gtexportal.org/home/datasets) for analysis, respectively. The whole data were filtered to remove missing and duplicated results, and transformed by log2(TPM +1) using R package of “rma” in an R environment (R version: 3.6.1). Corresponding patients’ records were also downloaded from the UCSC browser, and the cases without follow-up records were removed. The expression profiles of SLC41A3 in pan-cancer were presented with The Tumor Immune Estimation Resource (TIMER, https://cistrome.shinyapps.io/timer/) and Gene Expression Profiling Interactive Analysis (GEPIA, http://gepia.cancer-pku.cn/index.html) database (24, 25). Also, another unifying TCGA and GETx datasets were performed to evaluate the SLC41A3 expression profiles in pan-cancers (26). The RNA sequencing data and corresponding clinical information of LIHC patients were extracted from the ICGC-LIRI.

### Cox Regression Analysis and Kaplan-Meier Survival Analysis

Cox regression analysis was used to evaluate the relationship between SLC41A3 expression and overall survival and disease-specific survival of patients using the TCGA databases. The Kaplan-Meier method was used to assess the difference between “high” and “low” risk groups based on the best separation of SLC41A3 expression, employing R packages of survminer and survival. The “surv-cutpoint” function in the survminer R package was performed to search the best split by verifying all potential cut points. Subsequently, the patients were divided into high and low SLC41A3 expression groups with the maximum-selected log-rank statistic. Log-rank *P*-value, hazard ratio (HR), and 95% confidence intervals were examined.

### SLC41A3 Expression Association With Immune Cells

The Tumor Immune Estimation Resource (TIMER) database was used to evaluate the correlation between SLC41A3 expression and immune cell infiltration (27). The TIMER database provided a crucial assessment and integration of immune cells for RNA sequencing samples from TCGA, including B cells, CD4+ T cells, CD8+ T cells, macrophages, neutrophils, and dendritic cells.

#### Integrated Network and Gene Set Enrichment Analysis

ComPPI, the compartmentalized protein-protein interaction database (https://comppi.linkgroup.hu/downloads), was used to explore the integrated network of SLC41A3. The Gene Set Enrichment Analysis (GSEA) software (http://www.broadinstitute.org/gsea) was used to perform pathway analysis. The enriched gene sets were selected based on a false discovery rate (FDR) < 0.05 and a family‐wise error rate <0.05.

#### Expression of SLC41A3 in Liver Hepatocellular Carcinoma

HCCDB (database of LIHC expression atlas) was used to evaluate the expression of SLC41A3 in LIHC, including GSE22058, GSE36376, GSE14520, GSE54236, GSE63898, GSE64041, GSE76427, and ICGC-LIRI (28). Meanwhile, the association between SLC41A3 expression and the clinical stage was assessed using the UALCAN database (29). To further evaluate the prognostic value of SLC41A3 in LIHC, we conducted the Cox regression analysis and the time-dependent receiver operating characteristic (ROC) curve analysis, respectively.

### Establishment and Evaluation of the Nomogram

In the present study, SLC41A3 expression and the clinical stage were used to build a nomogram, which is an effective and convenient approach for estimating the overall survival in individual patients (30). The calibration curve and ROC curve were performed to verify and estimate the prediction accuracy of the nomogram.

## Results

### Expression Levels of SLC41A3 in Pan-Cancer

**Figure 1:**
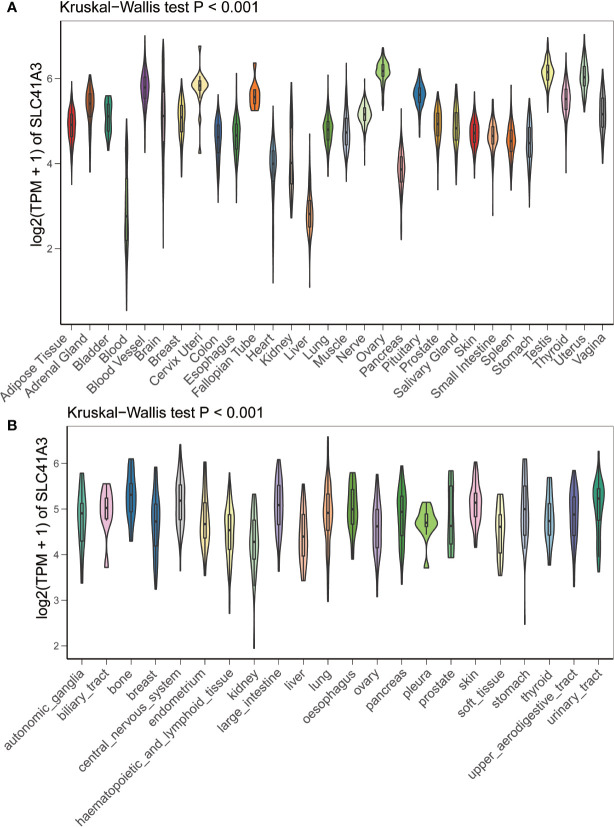
mRNA expression profile of SLC41A3. **(A)** SLC41A3 expression in normal tissue based on GETx database. **(B)** SLC41A3 expression in various tumor cell lines based on Cancer Cell Line Encyclopedia (CCLE) database. Kruskal-Wallis test was used to determine significant differences.

**Figure 2:**
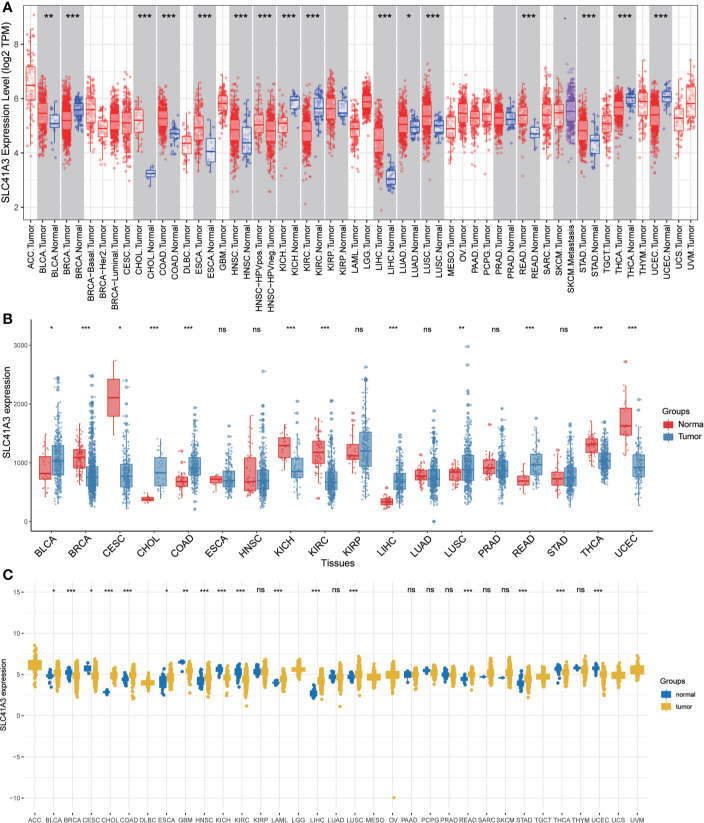
mRNA expression profile of SLC41A3 in The Cancer Genome Atlas (TCGA) cohorts. **(A)** Pan-cancer expression profile of SLC41A3 from TIMER database. **(B)** Increased or decreased expression of SLC41A3 in various cancer from the Wang, Q et al. databases. **(C)** The SLC41A3 expression in pan-cancers from UCSC database. *p<0.05, **p<0.01, ***p<0.001. ACC, adrenocortical carcinoma; BLCA, bladder urothelial carcinoma; BRCA, breast invasive carcinoma; CESC, cervical and endocervical cancers; CHOL, cholangiocarcinoma; COAD, colon adenocarcinoma; DLBC, lymphoid neoplasm diffuse large B-cell lymphoma; ESCA, esophageal carcinoma; GBM, glioblastoma multiforme; HNSC, head and neck squamous cell carcinoma; KICH, kidney chromophobe; KIRC, kidney renal clear cell carcinoma; KIRP, kidney renal papillary cell carcinoma; LAML, acute myeloid leukemia; LGG, brain lower grade glioma; LIHC, liver hepatocellular carcinoma; LUAD, lung adenocarcinoma; LUSC, lung squamous cell carcinoma; MESO, mesothelioma; OV, ovarian serous cystadenocarcinoma; PAAD, pancreatic adenocarcinoma; PCPG, pheochromocytoma and paraganglioma; PRAD, prostate adenocarcinoma; READ, rectum adenocarcinoma; SARC, sarcoma; SKCM, skin cutaneous melanoma; STAD, stomach adenocarcinoma; STES, stomach and esophageal carcinoma; TGCT, testicular germ cell tumors; THCA, thyroid carcinoma; THYM, thymoma; UCEC, uterine corpus endometrial carcinoma; UCS, uterine carcinosarcoma; UVM, uveal melanoma.

We analyzed the mRNA expression of SLC41A3 in normal tissues using the GTEx database. The results showed that SLC41A3 expressions were the lowest in the blood and liver tissue compared with other tissues, while the highest expressions of SLC41A3 were found in the testis, uterus and ovary tissues compared to other tissues (Kruskal-Wallis test *P*<0.001) (**Figure 1A**), and the *P*-value derived from Wilcox test was shown in **Supplement1.csv**. Also, the ANOVA test showed the same trend (**Figure S1A**). Subsequently, we evaluated the mRNA expression of SLC41A3 in twenty-two cancer cell lines from the CCLE database, and the results revealed that the expression levels of SLC41A3 varied significantly among cancer cell lines [Kruskal-Wallis test *P*<0.001 (**Figure 1B**) with ANONA test *P*<0.001 (**Figure S1B**)]. Those results were consistent with those calculated with the GTEx (https://commonfund.nih.gov/GTEx/) and CCLE (https://portals.broadinstitute.org/ccle/about) database. Furthermore, three different databases based on the TCGA cohorts were used to evaluate the mRNA expression of SLC41A3 in thirty-three cancer types. The results from the TIMER database showed that SLC41A3 expression was significantly increased in some cancers, including Bladder Urothelial Carcinoma (BLCA), cholangiocarcinoma (CHOL), Colon adenocarcinoma (COAD), Esophageal carcinoma (ESCA), Head and Neck squamous cell carcinoma (HNSC), LIHC, Lung adenocarcinoma (LUAD), Lung squamous cell carcinoma (LUSC), Rectum adenocarcinoma (READ) and stomach adenocarcinoma (STAD), compared with the normal tissues. However, decreased SLC41A3 expression was found in the Breast invasive carcinoma (BRCA), kidney chromophobe (KICH), kidney renal clear cell carcinoma (KIRC), uterine corpus endometrial carcinoma (UCEC), and thyroid carcinoma (THCA) compared to their corresponding adjacent non-cancerous tissues (**Figure 2A**). The results from Wang, Q et al. suggested that SLC41A3 was overexpressed in BLCA, CHOL, COAD, LIHC, LUSC and READ compared to the corresponding adjacent normal tissues. Whereas SLC41A3 expression was downregulated in BRCA, CESC, KICH, KIRC, THCA and UCEC compared with non-tumor tissue (**Figure 2B**). The SLC41A3 expression level retrieved from UCSC were basically consistent with the TIMER database (**Figure 2C**), and the SLC41A3 TPM expression was shown in Supplement 2.csv. The results from three different databases exhibited high SLC41A3 expressions in BLCA, CHOL, COAD, LIHC, LUSC, and READ, but low SLC41A3 expression in BRCA, KICH, KIRC, KICH, THCA, and UCEC compared with non-tumor tissue. The results from the TIMER and UCSC database indicated that SLC41A3 expression was significantly elevated in ESCA, HNSC, and STAD compared with the normal tissue, however, these differences were not statistically significant in Wang, Q et al. dataset.

### Prognostic Analysis of SLC41A3 in Pan-Cancer

**Figure 3:**
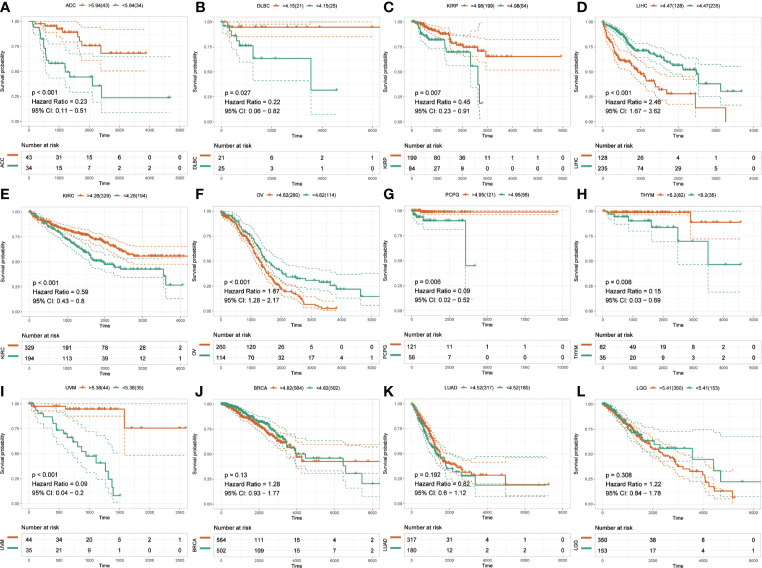
Overall survival (OS) of SLC41A3 in different cancer types. **(A)** Overall survival (OS) difference between groups in adrenocortical carcinoma (ACC). **(B)** OS difference between groups in lymphoid neoplasm diffuse large B-cell lymphoma (DLBC). **(C)** OS difference between groups in kidney renal papillary cell carcinoma (KIRP). **(D)** OS difference between groups in liver hepatocellular carcinoma (LIHC). **(E)** OS difference between groups in kidney renal clear cell carcinoma (KIRC). **(F)** OS difference between groups in ovarian serous cystadenocarcinoma (OV). **(G)** OS difference between groups in pheochromocytoma and paraganglioma (PCPG). **(H)** OS difference between groups in thymoma (THYM). **(I)** OS difference between groups in uveal melanoma (UVM). **(J)** OS difference between groups in breast invasive carcinoma (BRCA). **(K)** OS difference between groups in lung adenocarcinoma (LUAD). **(L)** OS difference between groups in brain lower grade glioma (LGG).

**Figure 4:**
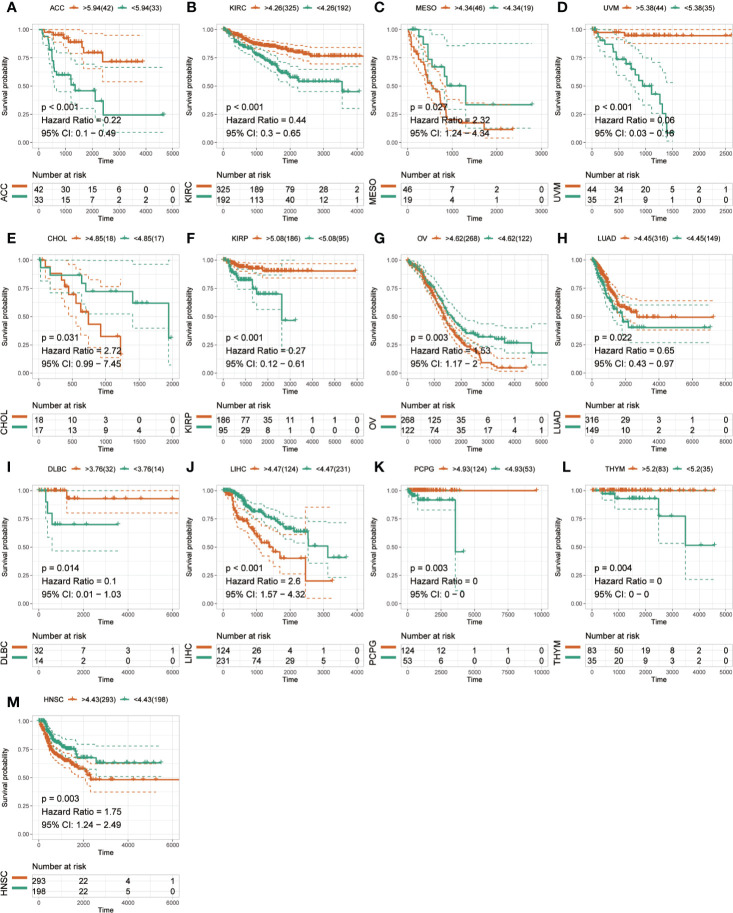
Disease-specific survival (DSS) of SLC41A3 in different cancer types. **(A)** DSS difference between groups in adrenocortical carcinoma (ACC). **(B)** DSS difference between groups in kidney renal clear cell carcinoma (KIRC). **(C)** DSS difference between groups in mesothelioma (MESO). **(D)** DSS difference between groups in uveal melanoma (UVM). **(E)** DSS difference between groups in cholangiocarcinoma (CHOL). **(F)** DSS difference between groups in kidney renal papillary cell carcinoma (KIRP). **(G)** DSS difference between groups in ovarian serous cystadenocarcinoma (OV). **(H)** DSS difference between groups in lung adenocarcinoma (LUAD). **(I)** DSS difference between groups in lymphoid neoplasm diffuse large B-cell lymphoma (DLBC). **(J)** DSS difference between groups in liver hepatocellular carcinoma (LIHC). **(K)** DSS difference between groups in pheochromocytoma and paraganglioma (PCPG). **(L)** DSS difference between groups in thymoma (THYM). **(M)** DSS difference between groups in head and neck squamous cell carcinoma (HNSC).

The Kaplan-Meier analysis was used to evaluate the association between SLC41A3 expression and overall survival in pan-cancer based on the TCGA database. The results revealed that increased SLC41A3 expression predicted better prognosis in ACC, DLBC, KIRP, KIRC, PCPG, THYM, and UVM, nevertheless, patients with low SLC41A3 expression showed remarkably good clinical outcome (OS) in LIHC and OV (**Figures 3A–I**). While, there was no prognostic relevance of SLC41A3 expression in some common tumors, including BRCA, LUAD, and LGG (**Figures 3J–L**, **Figures S2** and **S3**). Meanwhile, COX regression also indicated that SLC41A3 expression was associated with the overall survival of ACC, KIRC, KIRP, LIHC, PCPG, THYM, and UVM (**Figure S4**). Furthermore, we analyzed the relationship between SLC41A3 expression and disease-specific survival (DSS) in pan-cancer. The results of the Kaplan-Meier plot suggested that increased expression of SLC41A3 was associated with poor DSS in MESO, CHOL, OV, LIHC, and HNSC, whereas, increased SLC41A3 expression predicted good DSS in ACC, KIRC, UVM, KIRP, LUAD, DLBC, PCPG, and THYM (**Figure 4**). However, SLC41A3 expression presented no impact on patients DSS in BLCA, BRCA, CESC, COAD, and LGG (**Figures S5** and **S6**). The results of COX regression analysis showed that SLC41A3 expression was associated with DSS in ACC, HNSC, KIRC, KIRP, LIHC, PCPG, THYM, and UVM (**Figure S7**). Notably, the SLC41A3 expression was distinctly associated with the DSS in ACC, KIRC, THYM, LIHC, and UVM. More detailed information about aberrant SLC41A3 expression and clinical outcome (OS and DSS) were shown in **Table S1**.

### SLC41A3 Expression and Immune Cells Infiltration Analyses

**Figure 5:**
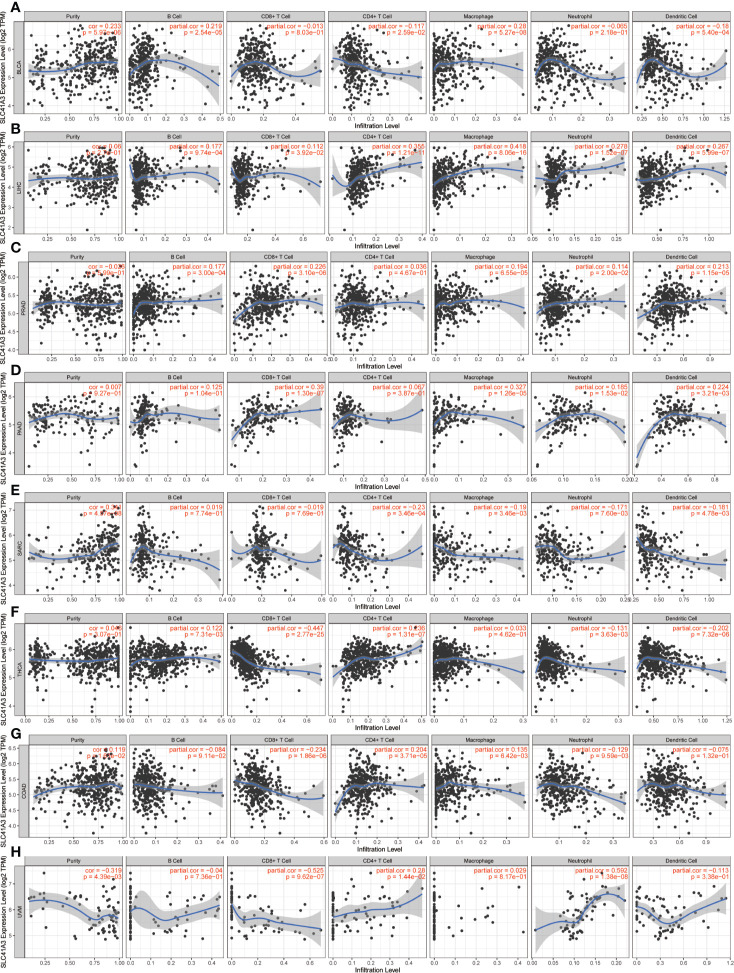
Correlation between SLC41A3 expression level and immune cell infiltration. Correlation between six immune cell infiltration scores (B cell, CD4+ T cell, CD8+ T cell, neutrophil, macrophage, dendritic cell) and SLC41A3 mRNA expression in **(A)** bladder urothelial carcinoma (BLCA), **(B)** liver hepatocellular carcinoma (LIHC), **(C)** prostate adenocarcinoma (PRAD), **(D)** pancreatic adenocarcinoma (PAAD), **(E)** sarcoma (SARC), **(F)** thyroid carcinoma (THCCA), **(G)** colon adenocarcinoma (COAD), and **(H)** uveal melanoma (UVM). (Spearman correlation test, *p*< 0.05 was considered significant).

Although previous results support prognostic implications of SLC41A3 in different cancers, its potential role warranted additional investigations. Immune cells play a crucial role in the immune microenvironment and can affect the prognosis of patients with cancer. It is unclear whether SLC41A3 impacts the recruitment of immune cells. Here, we evaluated the correlation between the immune cell infiltration and SLC41A3 expression in pan-cancer based on the TIMER database. The score of six immune cell types, including B cells, CD4+ T cells, CD8+ T cells, neutrophils, macrophages, and dendritic cells, were calculated based on the TCGA database. We found increased B cells infiltration were associated with increased SLC41A3 expression in the following cancers: BLCA, LIHC, PRAD, THCA, ESCA, HNSC, LUAD, LUSC, and PCPG. In addition, elevated SLC41A3 expression were also associated with increased level of CD4+ T cells, CD8+ T cells, neutrophils, and dendritic cells infiltration in BLCA, LIHC, PRAD, PAAD, SARC, THCA, COAD, UVM, CESC, ESCA, GBM, HNSC, KIRC, KIRP, LGG, LUAD, LUSC, MESO, and STAD. The infiltration of all these immune cells showed a significant correlation with SLC41A3 expression in LIHC. In contrast, no correlation was found between SLC41A3 expression and immune cell infiltration in ACC, BRCA, CHOL, UCS, DLBC, KICH, OV, TGCT, and THYM. The results were displayed in **Figure 5** and **Figures S8**-**10**.

**Figure 6:**
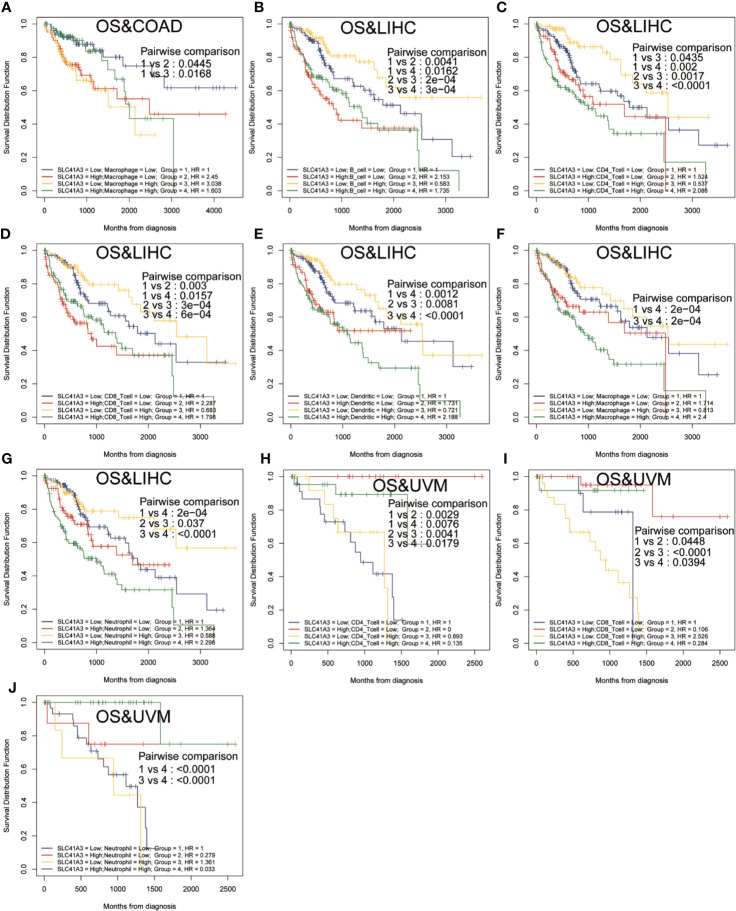
Overall survival (OS) curves using combinations SLC41A3 expression and Immune cells score. **(A)** OS analysis of combinations SLC41A3 expression and Macrophage cell score in colon adenocarcinoma (COAD). **(B)** OS analysis of combinations SLC41A3 expression and B cell score in liver hepatocellular carcinoma (LIHC). **(C)** OS analysis of combinations SLC41A3 expression and CD4+ T cell score in LIHC. **(D)** OS analysis of combinations SLC41A3 expression and CD8+ T cell score in LIHC. **(E)** OS analysis of combinations SLC41A3 expression and dendritic cell score in LIHC. **(F)** OS analysis of combinations SLC41A3 expression and macrophage cell score in LIHC. **(G)** OS analysis of combinations SLC41A3 expression and neutrophil cell score in LIHC. **(H)** OS analysis of combinations SLC41A3 expression and CD4+ T cell score in uveal melanoma (UVM). **(I)** OS analysis of combinations SLC41A3 expression and CD8+ T cell score in UVM. **(J)** OS analysis of combinations SLC41A3 expression and Neutrophil cell score in UVM. *p*< 0.05 was considered significant.

**Figure 7:**
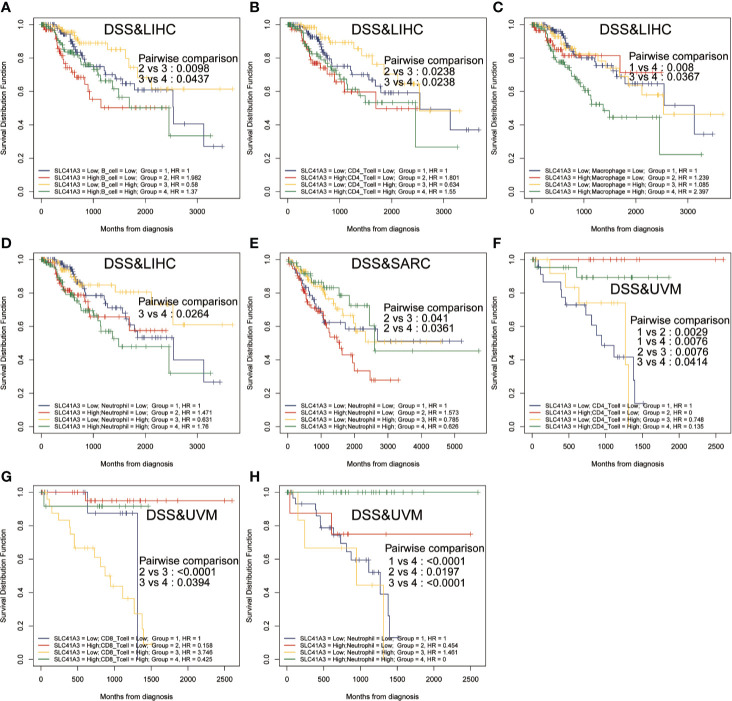
Disease-specific survival (DSS) curves using combinations SLC41A3 expression and Immune cells score. **(A)** DSS analysis of combinations SLC41A3 expression and B cell score in liver hepatocellular carcinoma (LIHC). **(B)** DSS analysis of combinations SLC41A3 expression and CD4+ T cell score in LIHC. **(C)** DSS analysis of combinations SLC41A3 expression and Macrophage cell score in LIHC. **(D)** DSS analysis of combinations SLC41A3 expression and Neutrophil cell score in LIHC. **(E)** DSS analysis of combinations SLC41A3 expression and neutrophil cell score in sarcoma (SARC). **(F)** DSS analysis of combinations SLC41A3 expression and CD4+ T cell score in uveal melanoma (UVM). **(G)** DSS analysis of combinations SLC41A3 expression and CD8+ T cell score in UVM. **(H)** DSS analysis of combinations SLC41A3 expression and neutrophil cell score in UVM. *p*< 0.05 was considered significant.

Furthermore, Kaplan-Meier analysis was performed to detect the correlation between the aberrant SLC41A3 expression and immune cell infiltration with clinical performance. Combined SLC41A3 expression with Macrophage cells score analysis indicated that COAD patients with low expression of SLC41A3 and macrophage presented the best overall survival (OS) (**Figure 6A**). The low SLC41A3 expression level and high scores of B cell, CD4+ T cell, CD8+ cell, macrophage, dendritic cell, or neutrophil cell showed the best OS in LIHC patients (**Figures 6B–G**). As expected, the groups with combined high SLC41A3 expression and low B cell score or low CD8+ T cell and high Macrophage cell score showed the worst OS in LIHC cohort. It is well known that tumor associated macrophages were negatively correlated with clinical outcome and B cell, CD4+ T cell, CD8+ cell, neutrophil cell, and dendritic cell were positively correlated with prognosis. Since low SLC41A3 expression was associated with better OS in UVM, therefore, the group with combined low SLC41A3 expression and low CD4+ T cell or low CD8+ T cell or low neutrophil cell presented worse OS (**Figures 6H–J**). Meanwhile, LIHC patients with low SLC41A3 expression and high B cell score, CD4+ T cell score or high neutrophil cell score had better DSS, whereas those with low SLC41A3 expression and low macrophage score presented better DSS (**Figures 7A–D**). SARC patients with high SLC41A3 expression and low Neutrophil score had worst DSS (**Figure 7E**). UVM patients with low SLC41A3 expression and low CD4+ T cell score were associated with worst DSS (**Figure 7F**). Additionally, UVM patients with high SLC41A3 expression had better DSS than those with low SLC41A3 expression, regardless of low CD8+ cell or neutrophil scores (**Figures 7G, H**). The details are shown in **Figures S11**-**S14**.

### Tumor Mutation Burden and Microsatellite Instability, Immune Checkpoint, Mismatch Repair, DNA Methyltransferase Analyses

**Figure 8:**
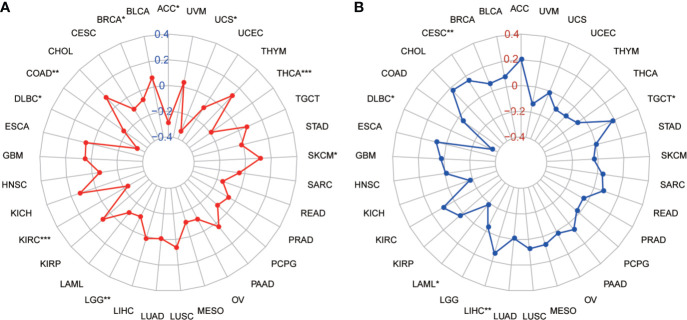
Correlation of SLC41A3 expression with tumor mutation burden (TMB) and microsatellite instability (MSI) in multiple cancer. **(A)** Correlation between TMB and SLC41A3 expression. **(B)** Correlation between MSI and SLC41A3 expression. Spearman’s correlation coefficients are shown above the bar graphs. (Spearman Correlation test, *p*< 0.05 was considered significant, *p<0.05, **p<0.01, ***p<0.001.).

**Figure 9:**
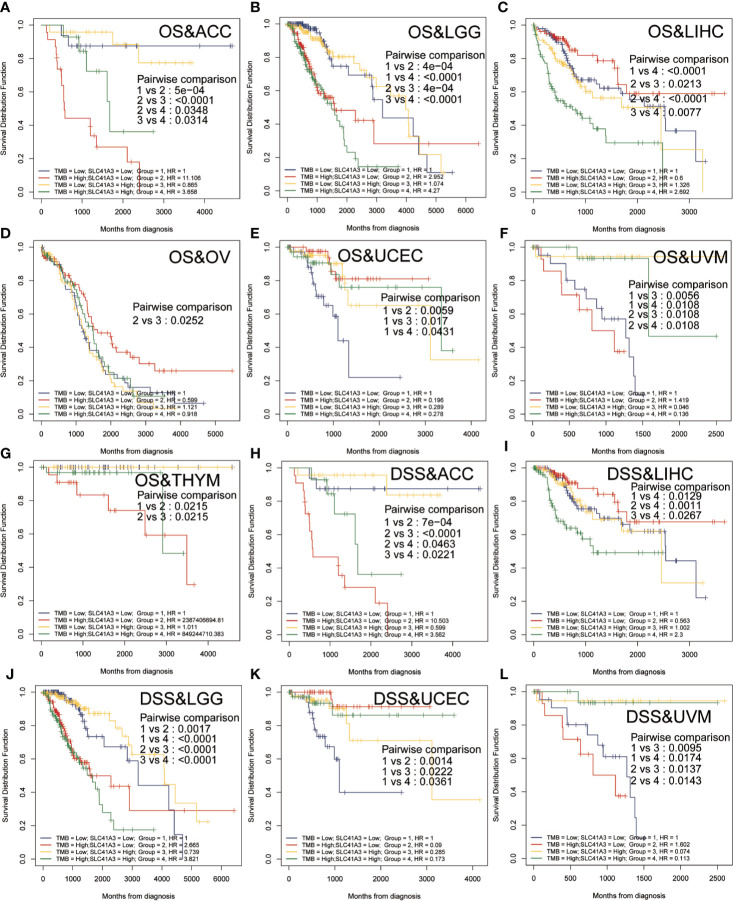
Overall survival (OS) and disease-specific survival (DSS) analysis combinations SLC41A3 expression and tumor mutation burden (TMB). **(A)** OS analysis of combinations SLC41A3 expression and TMB in adrenocortical carcinoma (ACC). **(B)** OS analysis of combinations SLC41A3 expression and TMB in brain lower grade glioma (LGG). **(C)** OS analysis of combinations SLC41A3 expression and TMB in liver hepatocellular carcinoma (LIHC). **(D)** OS analysis of combinations SLC41A3 expression and TMB in ovarian serous cystadenocarcinoma (OV). **(E)** OS analysis of combinations SLC41A3 expression and TMB in uterine corpus endometrial carcinoma (UCEC). **(F)** OS analysis of combinations SLC41A3 expression and TMB in uveal melanoma (UVM). **(G)** OS analysis of combinations SLC41A3 expression and TMB in thymoma (THYM). **(H)** DSS analysis of combinations SLC41A3 expression and TMB in ACC. **(I)** DSS analysis of combinations SLC41A3 expression and TMB in LIHC. **(J)** DSS analysis of combinations SLC41A3 expression and TMB in LGG. **(K)** DSS analysis of combinations SLC41A3 expression and TMB in UCEC. **(L)** DSS analysis of combinations SLC41A3 expression and TMB in UVM. *p*< 0.05 was considered significant.

**Figure 10:**
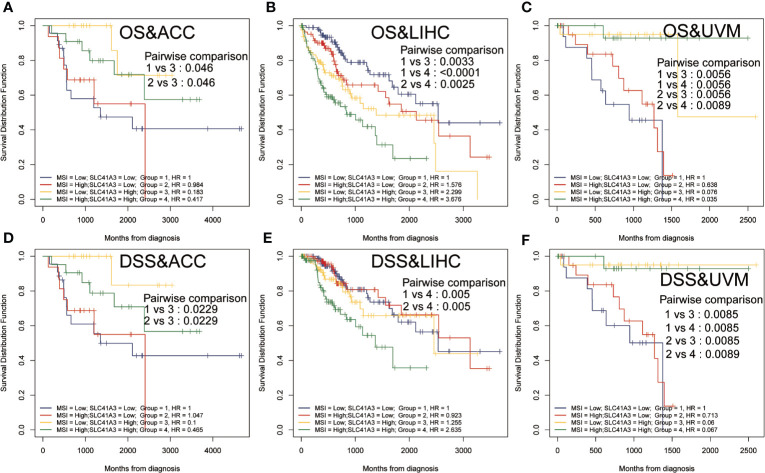
Overall survival (OS) and disease-specific survival (DSS) analysis combinations SLC41A3 expression and microsatellite instability (MSI). **(A)** OS analysis of combinations SLC41A3 expression and MSI in adrenocortical carcinoma (ACC). **(B)** OS analysis of combinations SLC41A3 expression and MSI in liver hepatocellular carcinoma (LIHC). **(C)** OS analysis of combinations SLC41A3 expression and MSI in uveal melanoma (UVM). **(D)** DSS analysis of combinations SLC41A3 expression and MSI in ACC. **(E)** DSS analysis of combinations SLC41A3 expression and MSI in LIHC. **(F)** DSS analysis of combinations SLC41A3 expression and MSI in UVM. *p*< 0.05 was considered significant.

Tumor mutation burden (TMB) and microsatellite instability (MSI) are considered essential factors impacting on the occurrence and progression of tumor. We analyzed the correlation between TMB or MSI and SLC41A3 expression in 33 common cancers to explore the relationship between the SLC41A3 activity and mutation in pan-cancer. The results demonstrated that decreased SLC41A3 expression was significantly associated with decreased TMB in ACC, BRCA, COAD, DLBC, KIRC, LGG, and UCS, while, increased SLC41A3 expression was positively associated with TMB in SKCM and THCA (**Figure 8A**). A significant correlation was found between increased SLC41A3 expression and increased MSI in various cancers, such as CESC, LIHC, and TGCT, while, decreased SLC41A3 expression was inversely correlated with MSI in DLBC and LAML (**Figure 8B**). The detailed information is shown in **Table S2**. Furthermore, we found that ACC patients with high TMB and low SLC41A3 expression had worst OS, while those with low TMB level and high SLC41A3 showed the best OS (**Figure 9A**). LGG patients with high TMB level had worse OS than those with low TMB, despite high SLC41A3 expression, this suggests SLC41A3 expression alone is insufficient to affect the clinical outcome in these patients (**Figure 9B**). LIHC patients with high SLC41A3 expression and high TMB level had the worst OS, in the meantime, patients with low SLC41A3 expression, or low TMB had better OS (**Figure 9C**). OV patients with high TMB level and low SLC41A3 expression had better OS, than those OV patients with low TMB and high SLC41A3 expression (**Figure 9D**). UCEC patients with low TMB level and low SLC41A3 expression had the worst OS (**Figure 9E**). UVM patients with low SLC41A3 expression had worse OS than those with high SLC41A3 expression, regardless of TMB level (**Figure 9F**). This may indicate that SLC41A3 level has more impact on the clinical in UVM patients. THYM patients with high TMB level and low SLC41A3 expression had the worst OS, patients with low TMB and high SLC41A3 expression presented the best OS (**Figure 9G**). The impact of SLC41A3 and TMB on DSS in ACC and LIHC cohort were almost consistent with OS (**Figures 9H, I**). LGG patients with high TMB level had worse DSS than those with low TMB level, regardless of high SLC41A3 expression, probably because SLC41A3 alone had no effect on clinical outcome (**Figure 9J**). UCEC patients with low TMB level and low SLC41A3 expression had the worst DSS (**Figure 9K**). UVM patients with high TMB level and low SLC41A3 expression had the worst DSS, meanwhile, the patients with low SLC41A3 expression presented worse DSS than those with high SLC41A3 expression, regardless of TMB level (**Figure 9L**). ACC patients with low SLC41A3 expression had worse OS/DSS than those with high SLC41A3 expression, regardless of MSI level, probably because MSI alone had no effect on clinical outcome (**Figures 10A, D**). As expected, LIHC patients with high SLC41A3 expression and high MSI level showed worse OS/DSS (**Figures 10B, E**). UVM patients with low SLC41A3 expression had worse OS/DSS than those with high SLC41A3 expression, regardless of MSI level, probably because MSI alone had no effect on clinical outcome (**Figures 10C, F**). The details are shown in **Figures S15**-**S18**.

The application of cancer immunotherapy has gained great attention in recent years. Therefore, we selected more than 40 common immune checkpoint genes from the previous data, and the association between SLC41A3 expression and the expression of immune checkpoint genes was evaluated (31–34). Interestingly, CD276 and CD200 expression showed a significant positive association with SLC41A3 expression in multiple cancers. In addition, SLC41A3 expression was associated with the levels of various immune checkpoints in THCA, TGCT, and LIHC (**Figure S19A**). Mismatch repair (MMR) is an intracellular process in which loss of function of key genes can lead to DNA replication errors that cannot be repaired. Hence, we investigated the correlation between SLC41A3 expression and four MMR genes (*MLH1*, *MSH2*, *MSH6*, and *PMS2*) in pan-cancer based on the TCGA database. The results showed that aberrant expression of SLC41A3 was significantly associated with the mutations of MMR genes in BLCA, BRCA, CESC, HNSC, KIRC, KIRP, LGG, LIHC, PRAD, THCA, THYM, and UCEC (**Figure S19B**). DNA methylation is believed to play an important role in epigenetics. In order to identify the correlation between SLC41A3 expression and DNA methylation, we analyzed the association between SLC41A3 expression and four methylation transferases (DNMT1, DNMT2, DNMT3A, and DNMT3B) in the present study. The results illustrated co-expression in multiple cancers such as, STAD, TGCT, THCA, THYM, UCEC, BLCA, BRCA, COAD, ESCA, HNSC, KIRC, KIRP, LAML, LIHC, LUSC, OV, PAAD, PCPG, PRAD, and SKCM. Especially, LIHC and THYM indicated high co-expression coefficients (about 0.4) in four methylation transferases (**Figure S20**).

### Further Validation in Liver Hepatocellular Carcinoma

**Figure 11:**
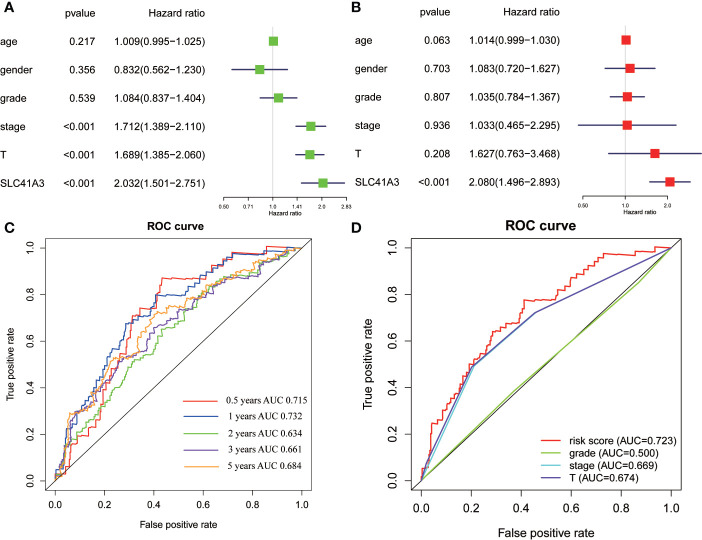
Cox regression and receiver operating characteristic (ROC) curves analysis. Univariate analysis **(A)** and multivariate analysis **(B)** of overall survival in The Cancer Genome Atlas (TCGA) cohorts. **(C)** ROC curves showed the predictive efficiency of the SLC41A3 for hepatocellular carcinoma (HCC) patients based on TCGA cohorts. **(D)** ROC curves with respect to SLC41A3, grade, stage, and T-stage in the TCGA cohorts.

In the above analysis, we discovered a correlation between SLC41A3 expression and prognosis of multiple tumors and immune cells infiltration, and we also identified the relationship between SLC41A3 expression and MMR, DNMT, TMB, and MSI, which were significantly associated with LIHC. To further understand the prognostic role of SLC41A3 in LIHC, subsequently, univariate and multivariate Cox regression analyses were used to examine whether SLC41A3 is an independent prognostic factor. The results indicated that tumor-stage (P<0.001), tumor-T stage (P<0.001), and SLC41A3 (P<0.001) were associated with overall survival in the TCGA cohort (**Figure 11A**). Importantly, multivariate Cox regression analysis further revealed that SLC41A3 (P<0.001) was an independent risk factor for LIHC (**Figure 11B**). These findings, including clinical correlation and prognostic value, were verified in the ICGC cohort (**Figure S22**). Next, the time-dependent ROC curve analysis was performed to measure the potential of SLC41A3 as prognostic marker in LIHC. The area under the curve (AUC) values for 0.5-, 1-, 2-, 3-, and 5-years overall survival were 0.715, 0.732, 0.634, 0.661, and 0.684, respectively (**Figure 11C**). SLC41A3 showed a strong predictive power for 1-year survival in LIHC (**Figure 11D**). To further verify and evaluate the prognostic value of SLC41A3in LIHC, additional data were collected for expression validation and prognostic analysis. As expected, the levels of SLC41A3 were increased in LIHC compared with the normal tissues, based on the multiple GEO (GSE22058, GSE36376, GSE14520, GSE54236, GSE63898, GSE64041, GSE76427) and ICGC (ICGC-LIRI) datasets from HCCDB (**Figure S21A**). Furthermore, the UALCAN database was used to assess the association between SLC41A3 expression and clinical characteristics. The results demonstrated that the SLC41A3 expression levels were increased with tumor-stage and tumor-grade, but showed opposite results with respect to tumor weight. Meanwhile, the SLC41A3 expression level was increased in the Asian race and the TP53-mutant group compared with the Caucasian race and the TP53 wildtype group, respectively, and no difference with respect to gender, age and nodal metastasis was observed (**Figures S21B–I**).

### Analysis of Protein-Protein Interactions and Gene Set Enrichment Analysis

We explored the difference in the DNA methylation of SLC41A3 between LIHC and normal tissues based on the UALCAN database. The results showed that the DNA methylation level of SLC41A3 was significantly lower in LIHC, compared with that in the normal tissues. This was possibly the major cause of high expression of SLC41A3 in the tumor tissue (**Figure S23A**). Then, we constructed a protein-protein interaction network to determine proteins that can interact with the SLC41A3 protein based on the ComPPI database, and found that IGFBP5, TMBIM6, APP, and CREB3 were candidate proteins that could interact with SLC41A3 (**Figure S23B**). Finally, to explore the biological function of SLC41A3 in LIHC, GSEA was used to identify enriched pathways. The results showed that the high expression SLC41A3 group was significantly enriched with pathways associated with tumorigenesis, including cell-cycle, NOTCH signaling pathway, ADHERENS junction, WNT signaling pathway, pathways in cancer, and TGF beta signaling pathway (**Figures S23C–H**).

### Construction and Evaluation of Nomogram

**Figure 12:**
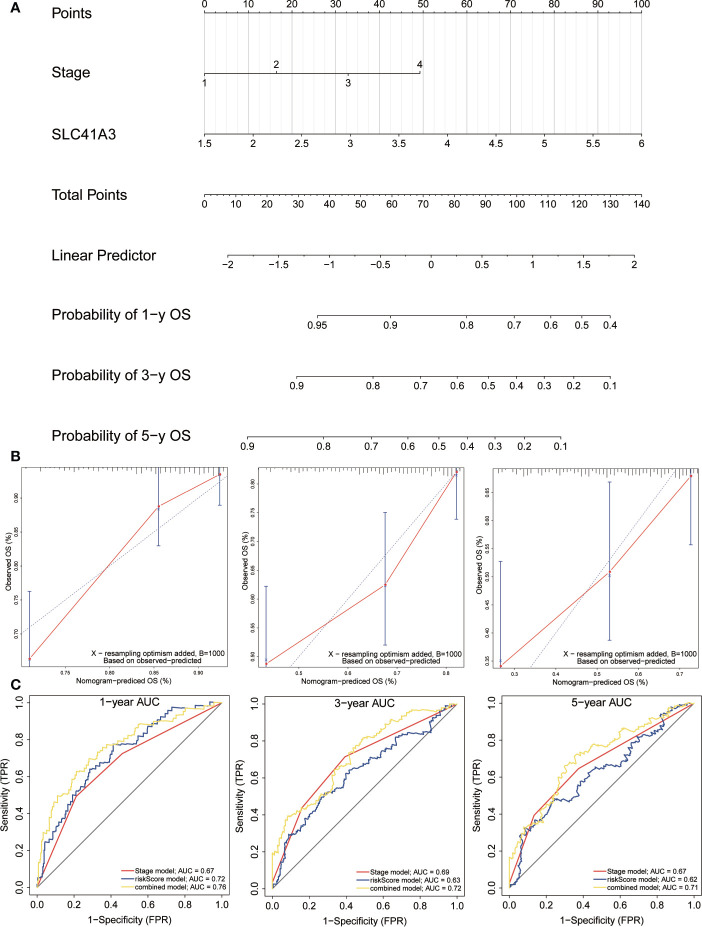
Construction and evaluation of nomogram. **(A)** The nomogram is applied by adding up SLC41A3 TPM value and stage plot. The total points projected on the bottom scales implied the probability 1-, 3-, 5-years overall survival. **(B)** Calibration curves of the nomogram for the prediction of survival rates at 1-, 3-, 5- year. The nomogram-predicted probability of survival is plotted on the x-axis; and the actual survival is plotted on the y-axis. **(C)** ROC curve analysis evaluates the accuracy of the nomograms.

In order to investigate the application of SLC41A3 in cancer prognosis, we built a nomogram for predicting the overall survival of LIHC patients in the TCGA cohort. The cancer stage and SLC41A3 were included as prognostic factors in the nomogram (**Figure 12A**). The calibration curve showed that the nomogram was reliable in predicting possibility of 1-, 3-, 5-years overall survival in LIHC (**Figure 12B**). The black line stand for ideal prediction, and the red line represents the actual fit. Meanwhile, the ROC analysis was performed to assess the prediction accuracy of the nomogram. The 1-, 3-, and 5-years AUC values were 0.73, 0.72, and 0.7, respectively (**Figure 12C**). Additionally, the nomogram showed the largest AUC at all time point compared to SLC41A3 and stage model. These results demonstrated that the nomogram combining SLC41A3 expression and clinical Stage had better predictive power for the overall survival of LIHC patients, which might contribute to efficacy assessment and managing patients.

## Discussion

Mg^2+^ is an essential element that bind to various protein and participate in a wide range of cellular function, such as genome stability and immune function (35, 36). Meanwhile, Mg^2+^ homeostasis is important in maintaining muscle contraction and blood pressure (37). Aberrant Mg^2+^ level have been found in various cancers and may facilitate cancer progression (38–41). SLC41A3 is a mitochondrial bound Mg^2+^ channel that participates in maintaining Mg^2+^ homeostasis, however, its aberrant expression in cancer is not well studied. Xie *et al*. reported that overexpression of the Mg2+ transporter protein SLC41A1 could facilitate Mg2+ efflux and could suppress tumor growth in pancreatic ductal adenocarcinoma (42).

In this study, we analyzed the pan cancer expression of SLC41A3 and the association of its aberrant expression with patient performance. Our results demonstrated that the expression of SLC41A3 was altered in various cancers and can affect cancer patient prognosis. We found SLC41A3 expression was significantly up-regulated in LIHC and CHOL, compared to the adjacent normal tissues, whereas the expression of SLC41A3 was down-regulated in CESC, OV, TGCT, and UCS. Therefore, it is possible that SLC41A3 may serve different functions in different types of cancers. COX regression and Kaplan-Meier analyses suggested an association between high SLC41A3 expression and poor OS and DSS in LIHC and KIRC. This result is consistent with our previous study that showed SLC41A3 is a prognostic factor in LIHC patients (43). Aberrant SLC41A3 expression may exert impact on cancer progression, in part, through influence on mechanisms that maintaining genome stability. Indeed, we found increase SLC41 expression and increased MSI in LIHC, and increase TMB in LGG and LIHC. It is worth noting that the aberrant expression of SLC41A3 is associated with increased mutation in MMR genes in UCEC. While we have yet to establish a cause-result relationship here, aberrant expression of SLC41A3 could conceivably synergize with MMR deficiency to promote tumor progression.

Mg^2+^ homeostasis may also influence immune function, such as immune cell adherence and macrophage response, and aberrant expression of SLC41A3 may alter cancer microenvironment and immune response, thus the overall clinical outcome (44–46). In the present study, we confirmed that aberrant SLC41A3 expression was associated with increased immune cells infiltration of CD4+ T cells, macrophages, and dendritic cells in BLCA, PRAD, PAAD, LIHC, and SARC. Combined SLC41A3 expression and immune cell score has more prognostic power in COAD, LIHC, and UVM, especially in LIHC. In addition, we also observed positive correlation between immune checkpoint gene and SLC41A3 expression in pan-cancer. Taking together, these results suggest aberrant SLC41A3 expression may alter tumor immune microenvironment.

In this study, we observed the most robust relationship of aberrant SLC41A3 expression with patient prognosis in LIHC. Consistent with our previous study, COX regression analysis using an external data set suggest SLC41A3 is an independent prognostic factor for LIHC patients, ROC suggest the predictability for SLC41A3 was excellent. This finding may be clinically useful in prognosis assessment and follow-up management of LIHC. Current, the precise mechanism of aberrant SLC41A3 expression in promoting LIHC progression is unclear. In addition to the PPI network analysis suggest SLC41A3 may directly interact with factors that promote cell growth, such as IGFBP5, TMBIM6, APP, and CREB3. Previous studies have reported that IGFBP5, TMBIM6, and CREB3 were involved in the development of LIHC (47–50). Alternatively, it may also activate pathways involved in tumorigenesis and immune response, such as NOTCH, WNT, and TGFB (51–53). These results pointed out potential roles of aberrant SLC41A3 expression in the initiation and development of LIHC.

The present study unveiled a complicated role of SLC41 aberrant expression in cancer progression and patient outcome that warrant further investigation. Since our study was based on bioinformatics and rely on public databases, there are major limitations. First, quality of data collection and the method used to generate the data could be inconsistent depend on the source. This could have impact on the conclusion of the some of the analysis. Secondly, the result and conclusions are not experimentally or prospectively validated in the laboratory or in clinic. Future study to validate the expression and function of SLC41A3 *in vivo* and *in vitro* is needed.

## Conclusion

SLC41A3 is differentially expressed in a variety of tumors and aberrant expression is associated with the progression of the tumor, especially in KIRC and LIHC. The aberrant SLC41A3 expression is associated with immune cells infiltration, immune checkpoint genes, MMR, DNMT, TMB, and MSI. Therefore, SLC41A3 may serve as a potential prognostic biomarker.

## Data Availability Statement

The original contributions presented in the study are included in the article/**Supplementary Material**. Further inquiries can be directed to the corresponding author.

## Author Contributions

JL, SZ, CX, and J-CL all took part in the design and data collection process of the study. JL, WD, and J-CL wrote the paper. All authors contributed to the article and approved the submitted version.

## Conflict of Interest

The authors declare that the research was conducted in the absence of any commercial or financial relationships that could be construed as a potential conflict of interest.

## Supplementary Material

Supplementary Figure 1 mRNA expression profile of SLC41A3. A. SLC41A3 expression in normal tissue based on GETx database. B. SLC41A3 expression in various tumor cell lines based on CCLE database. ANOVA test was used to determine significant differences.

Supplementary Figure 2 Overall survival (OS) of SLC41A3 in different cancer types. *p* < 0.05 was considered significant.

Supplementary Figure 3 Overall survival (OS) of SLC41A3 in different cancer types. *p* < 0.05 was considered significant.

Supplementary Figure 4 Relationship between expression of SLC41A3 and overall survival in pan-cancer. Cox regression was used to examine the correlation, *p*< 0.05 was considered significant.

Supplementary Figure 5 Disease-specific Survival (DSS) of SLC41A3 in different cancer types. *p*< 0.05 was considered significant.

Supplementary Figure 6 Disease-specific Survival (DSS) of SLC41A3 in different cancer types. *p*< 0.05 was considered significant.

Supplementary Figure 7 Relationship between expression of SLC41A3 and disease-specific survival in pan-cancer. Cox regression was used to examine the correlation, *p*< 0.05 was considered significant.

Supplementary Figure 8 Correlation between SLC41A3 expression level and immune cell infiltration. Correlation between six immune cell infiltration scores (B cell, CD4+ T cell, CD8+ T cell, Neutrophil, Macrophage, Dendritic cell) and SLC41A3 mRNA expression in (A) ACC, (B) BRCA, (C) CESC, (D) (CHOL), (E) UCS, (F) DLBC, (G) ESCA, (H) GBM.

Supplementary Figure 9 Correlation between SLC41A3 expression level and immune cell infiltration. Correlation between six immune cell infiltration scores (B cell, CD4+ T cell, CD8+ T cell, Neutrophil, Macrophage, Dendritic cell) and SLC41A3 mRNA expression in (A) HNSC, (B) KICH, (C) KIRC, (D) KIRP, (E) LGG, (F) LUAD, (G) LUSC, (H) MESO.

Supplementary Figure 10 Correlation between SLC41A3 expression level and immune cell infiltration. Correlation between six immune cell infiltration scores (B cell, CD4+ T cell, CD8+ T cell, Neutrophil, Macrophage, Dendritic cell) and SLC41A3 mRNA expression in (A) OV, (B) PCPG, (C) READ, (D) SKCM, (E) STAD, (F) TGCCT, (G) THYM, (H) UCEC.

Supplementary Figure 11 Overall Survival (OS) curves using combinations SLC41A3 expression and Immune cells score. p< 0.05 was considered significant.

Supplementary Figure 12 Overall Survival (OS) curves using combinations SLC41A3 expression and Immune cells score. p< 0.05 was considered significant.

Supplementary Figure 13 Disease-specific Survival (DSS) curves using combinations SLC41A3 expression and Immune cells score. p< 0.05 was considered significant.

Supplementary Figure 14 Disease-specific Survival (DSS) curves using combinations SLC41A3 expression and Immune cells score. p< 0.05 was considered significant.

Supplementary Figure 15 Overall survival (OS) analysis combinations SLC41A3 expression and tumor mutation burden (TMB). p< 0.05 was considered significant.

Supplementary Figure 16 Disease-specific Survival (DSS) analysis combinations SLC41A3 expression and tumor mutation burden (TMB). p< 0.05 was considered significant.

Supplementary Figure 17 Overall survival (OS) analysis combinations SLC41A3 expression and microsatellite instability (MSI). p< 0.05 was considered significant.

Supplementary Figure 18 Disease-specific Survival (DSS) analysis combinations SLC41A3 expression and microsatellite instability (MSI). p< 0.05 was considered significant.

Supplementary Figure 19 Relationship between immune checkpoints’ mRNA expression **(A)** and mismatch repair (MMR) defects **(B)** and SLC41A3 mRNA expression level in various tumors from TCGA database. The lower triangle in each tile indicates coefficients calculated by Spearman’s correlation test, and the upper triangle indicates log10 transformed p value. (*: p< 0.05, **: p<0.01, ***: p<0.001).

Supplementary Figure 20 Relationship between DNA methyltransferase (DNMT) and SLC41A3 mRNA expression level in various tumors from TCGA database. Red colors represented DNMT1, Blue colors represented DNMT2, Green colors represented DNMT3A, Purple colors represented DNMT3B. Spearman’s correlation test, p< 0.05 was considered significant.

Supplementary Figure 21 SLC41A3 expression and clinicopathologic characteristics analysis in HCC. A. SLC41A3 expression levels in HCC and normal tissues based on GEO and ICGC database., red for tumor, blue for normal. Association with the SLC41A3 expression and clinicopathologic characteristics, including Stages (B), Race (C), Gender (D), Age (E), Weight (F), Grade (G), Nodal metastasis status (H), Tp53 mutation status (I).

Supplementary Figure 22 Correlation between SLC41A3 expression and stage and prognosis in ICGC cohorts. A. SLC41A3 expression levels increased with advanced clinical stages. B. Overall survival analysis was examined with respect to SLC41A3 expression in ICGC cohorts. C. Univariate analysis of overall survival. D. Multivariate analysis of HCC patients in ICGC cohorts.

Supplementary Figure 23 The potential mechanisms and functions of SLC41A3. A. Promoter methylation level of SLC41A3 in HCC. B. ComPPI was performed to identify functionally relevant interactions of SLC41A3, the length of the line corresponds to the score of the interaction. KEGG pathway enrichment analysis of SLC41A3, including CELL cycle (C), NOTCH signaling pathway (D), ADHERENS junction (E), WENT signaling pathway (F), pathways in CANCER (G), TGF BETA signaling pathway (H).
