# Supplementary material for: A Comprehensive Prognostic and Immune Analysis of SLC41A3 in Pan-Cancer
Source: Front Oncol. 2021 Jan 14;10:586414. doi: 10.3389/fonc.2020.586414 (PMC7841432; doi:10.3389/fonc.2020.586414)
Supplement: Supplementary file 3 [file Table_1.docx]

Table S1. The relationships between SLC41A3 expression and both OS and DSS in pan-cancers.

| Tumor | expression level | OS.HR | OS. P-Value | DSS.HR | DSS.P-Value |
| --- | --- | --- | --- | --- | --- |
| ACC | NA | 0.23 | <0.001 | 0.22 | <0.001 |
| BLCA | High | 1.27 | 0.113 | 1.35 | 0.095 |
| BRCA | Low | 1.28 | 0.13 | 1.39 | 0.139 |
| CESC | Low | 0.82 | 0.417 | 1.3 | 0.341 |
| CHOL | High | 2.34 | 0.053 | 2.72 | 0.031 |
| COAD | High | 1.59 | 0.108 | 1.59 | 0.241 |
| DLBC | NA | 0.22 | 0.027 | 0.1 | 0.014 |
| ESCA | High | 0.82 | 0.446 | 1.26 | 0.421 |
| GBM | Low | 1.15 | 0.455 | 1.23 | 0.287 |
| HNSC | High | 1.2 | 0.197 | 1.75 | 0.003 |
| KICH | Low | 3.45 | 0.1 | 3.52 | 0.108 |
| KIRC | Low | 0.59 | <0.001 | 0.44 | <0.001 |
| KIRP | Ns | 0.45 | 0.007 | 0.27 | <0.001 |
| LAML | High | 1.21 | 0.425 | ns | ns |
| LGG | NA | 1.22 | 0.308 | 0.88 | 0.494 |
| LIHC | High | 2.46 | <0.001 | 2.6 | <0.001 |
| LUAD | Ns | 0.82 | 0.192 | 0.65 | 0.022 |
| LUSC | High | 1.19 | 0.219 | 0.68 | 0.078 |
| OV | NA | 1.67 | <0.001 | 1.53 | 0.003 |
| PAAD | Ns | 1.2 | 0.388 | 1.23 | 0.386 |
| PCPG | Ns | 0.09 | 0.006 | 0 | 0.003 |
| PRAD | Ns | 2.09 | 0.249 | 2.83 | 0.328 |
| READ | High | 1.65 | 0.291 | 2.3 | 0.348 |
| SARC | Ns | 1.37 | 0.116 | 1.33 | 0.203 |
| SKCM | Ns | 1.27 | 0.109 | 1.35 | 0.072 |
| STAD | High | 1.24 | 0.248 | 1.61 | 0.051 |
| TGCT | NA | 0 | 0.074 | 0 | 0.124 |
| THCA | Low | 0.59 | 0.289 | 2.12 | 0.314 |
| THYM | Ns | 0.15 | 0.008 | 0 | 0.004 |
| UCEC | Low | 0.52 | 0.059 | 0.5 | 0.1 |
| UCS | NA | 0.78 | 0.452 | 0.69 | 0.308 |
| MESO | NA | 1.54 | 0.076 | 2.32 | 0.027 |
| UVM | NA | 0.09 | <0.001 | 0.06 | <0.001 |

NA: not available. Ns: not significant

Table S2. Correlation of SLC41A3 expression with tumor mutation burden (TMB) and microsatellite instability (MSI) in multiple cancer.

| Cancer Type | Cor (TMB) | *P*-Value (TMB) | Cor (MSI) | *P*-Value (MSI) |
| --- | --- | --- | --- | --- |
| ACC | -0.28904 | 0.010788 | 0.202984 | 0.07664 |
| BLCA | 0.071993 | 0.147107 | 0.07833 | 0.11461 |
| BRCA | -0.07192 | 0.024291 | 0.060737 | 0.050321 |
| CESC | -0.10555 | 0.074724 | 0.159114 | 0.005583 |
| CHOL | 0.101507 | 0.555812 | 0.175032 | 0.305978 |
| COAD | -0.17418 | 0.003341 | -0.04146 | 0.485683 |
| DLBC | -0.33442 | 0.043069 | -0.3505 | 0.01572 |
| ESCA | 0.059461 | 0.427836 | 0.079982 | 0.285834 |
| GBM | 0.047812 | 0.555973 | 0.025908 | 0.747388 |
| HNSC | -0.06026 | 0.180314 | -0.00758 | 0.865766 |
| KICH | 0.125609 | 0.314926 | -0.17603 | 0.157412 |
| KIRC | -0.23685 | 1.22E-05 | 0.100782 | 0.064611 |
| KIRP | 0.072823 | 0.22531 | 0.031977 | 0.59085 |
| LAML | -0.1061 | 0.250764 | -0.18163 | 0.039396 |
| LGG | -0.12779 | 0.004171 | -0.0353 | 0.42727 |
| LIHC | 0.014927 | 0.778661 | 0.136711 | 0.008732 |
| LUAD | -0.00668 | 0.881193 | -0.00871 | 0.844292 |
| LUSC | 0.06304 | 0.165716 | 0.073489 | 0.104205 |
| MESO | -0.1169 | 0.301737 | 0.065059 | 0.558989 |
| OV | -0.10523 | 0.066455 | 0.019751 | 0.731187 |
| PAAD | 0.034023 | 0.677324 | 0.062692 | 0.40847 |
| PCPG | -0.09434 | 0.212994 | -0.02749 | 0.716465 |
| PRAD | -0.05972 | 0.190545 | -0.04251 | 0.345295 |
| READ | -0.15336 | 0.151329 | 0.07364 | 0.492823 |
| SARC | -0.04341 | 0.508729 | 0.037928 | 0.548971 |
| SKCM | 0.117342 | 0.01151 | -0.0351 | 0.449768 |
| STAD | -0.01507 | 0.762414 | -0.0035 | 0.943504 |
| TGCT | 0.070998 | 0.397768 | 0.178849 | 0.029637 |
| THCA | -0.19147 | 2.23E-05 | -0.06527 | 0.147843 |
| THYM | 0.120713 | 0.194826 | -0.10006 | 0.280983 |
| UCEC | -0.09149 | 0.228519 | -0.10721 | 0.151987 |
| UCS | -0.33673 | 0.010432 | -0.01643 | 0.903441 |
| UVM | 0.036137 | 0.75187 | -0.13672 | 0.229562 |
