# Supplementary figures and images for: A Comprehensive Prognostic and Immune Analysis of SLC41A3 in Pan-Cancer
Source: Front Oncol. 2021 Jan 14;10:586414. doi: 10.3389/fonc.2020.586414 (PMC7841432; doi:10.3389/fonc.2020.586414)

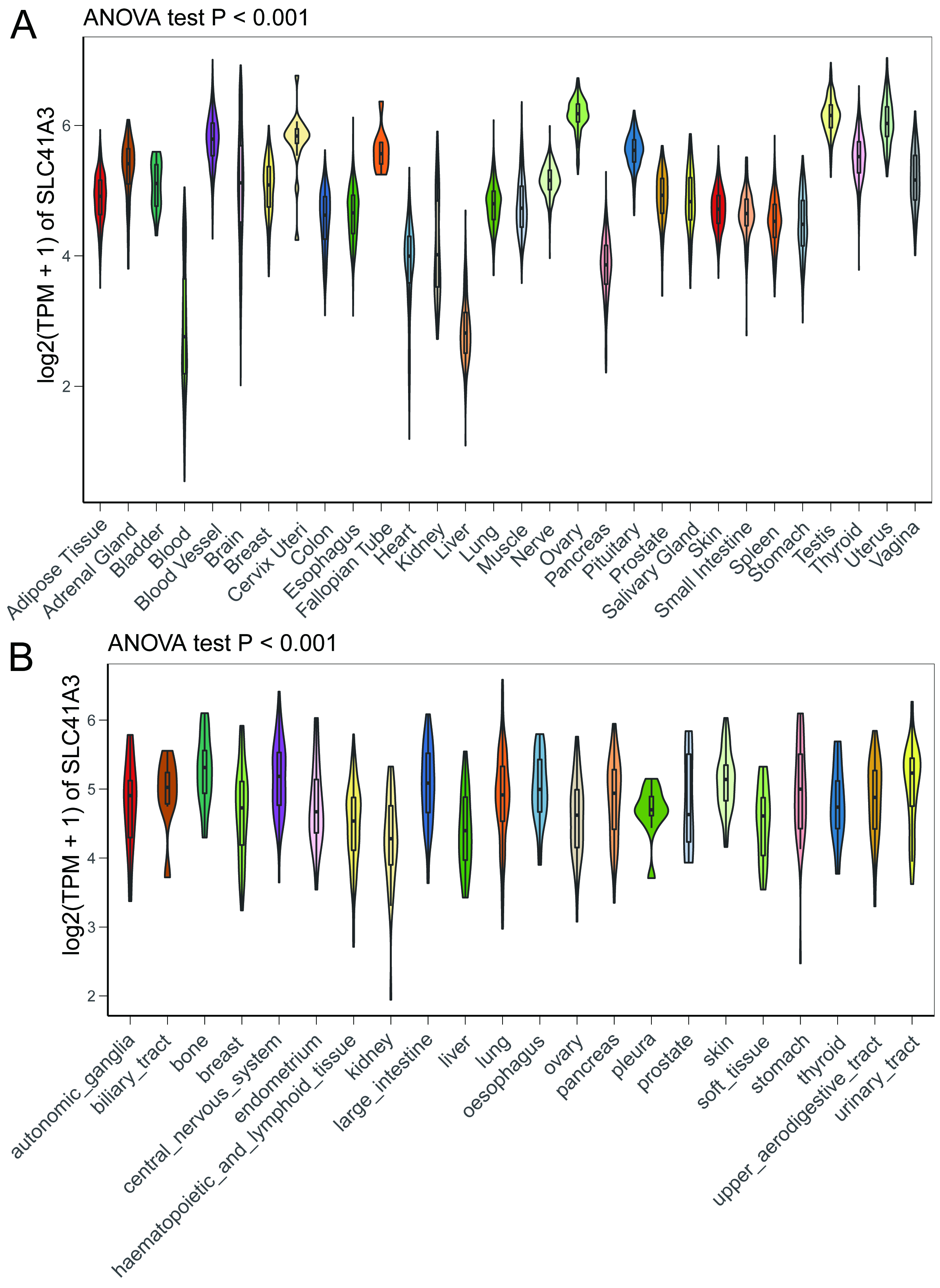

Supplement: Supplementary Figure 1 — mRNA expression profile of SLC41A3. A. SLC41A3 expression in normal tissue based on GETx database. B. SLC41A3 expression in various tumor cell lines based on CCLE database. ANOVA test was used to determine significant differences. [file Image_1.tif]

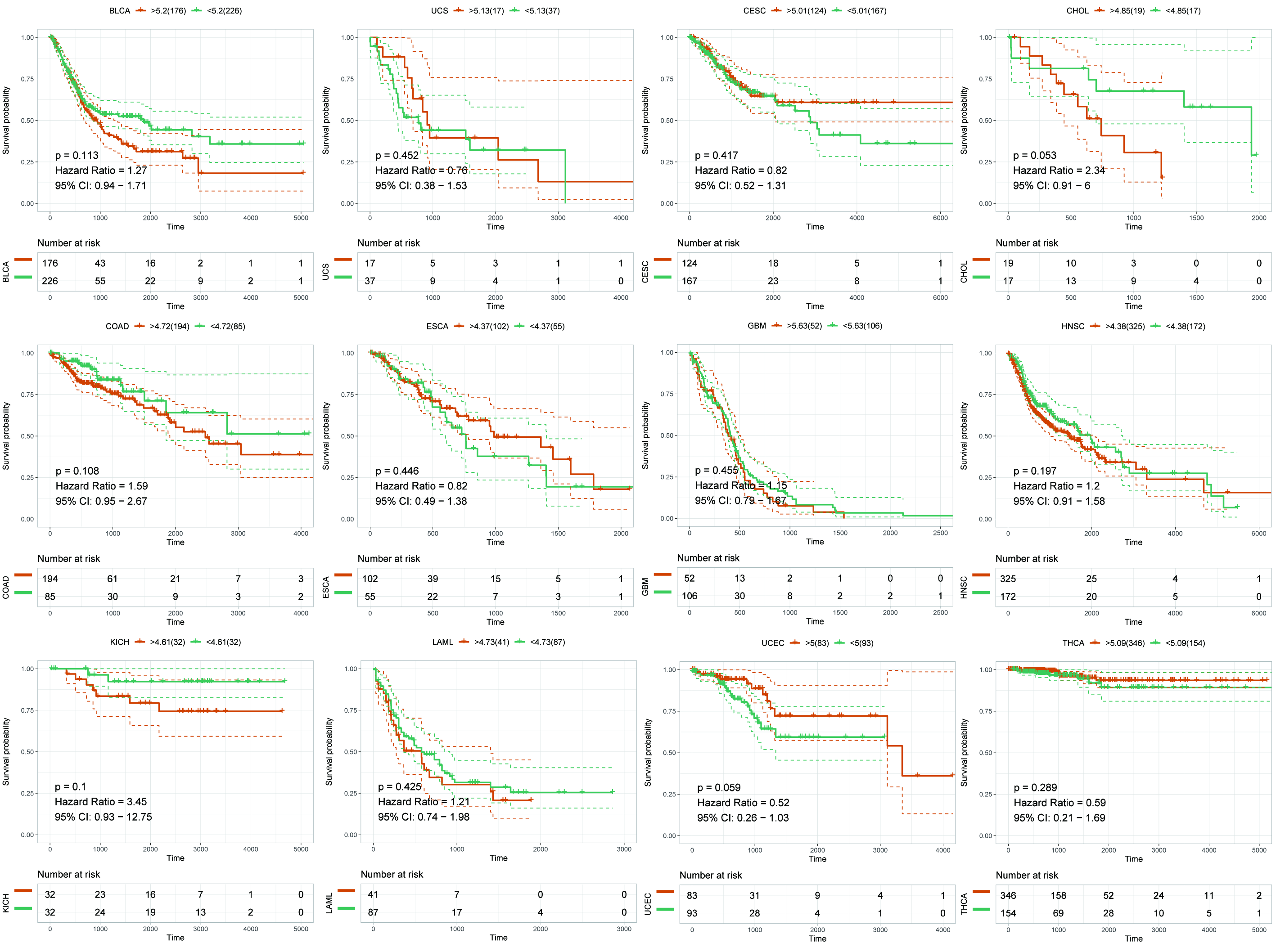

Supplement: Supplementary Figure 2 — Overall survival (OS) of SLC41A3 in different cancer types. p < 0.05 was considered significant. [file Image_2.tif]

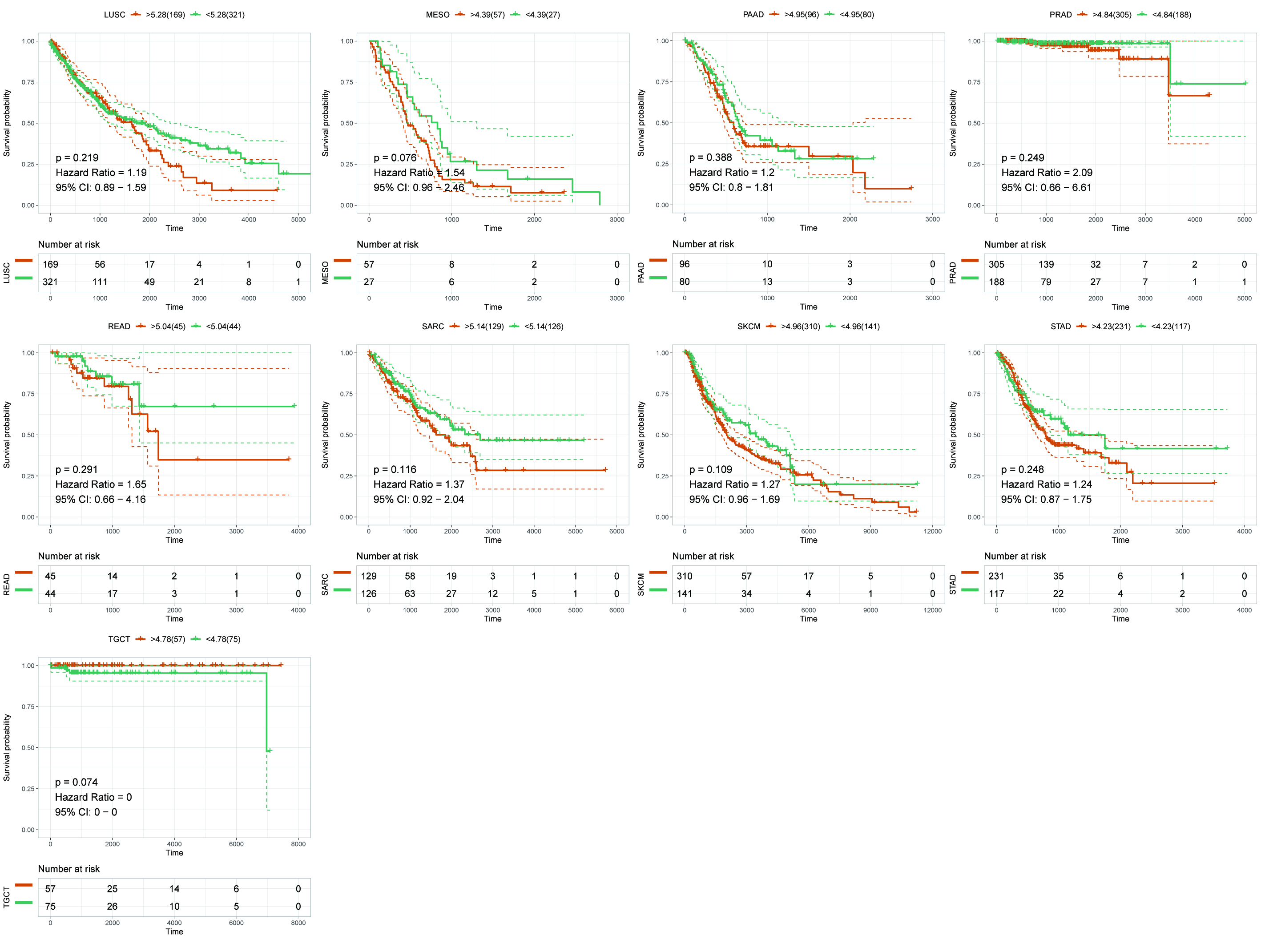

Supplement: Supplementary Figure 3 — Overall survival (OS) of SLC41A3 in different cancer types. p < 0.05 was considered significant. [file Image_3.tif]

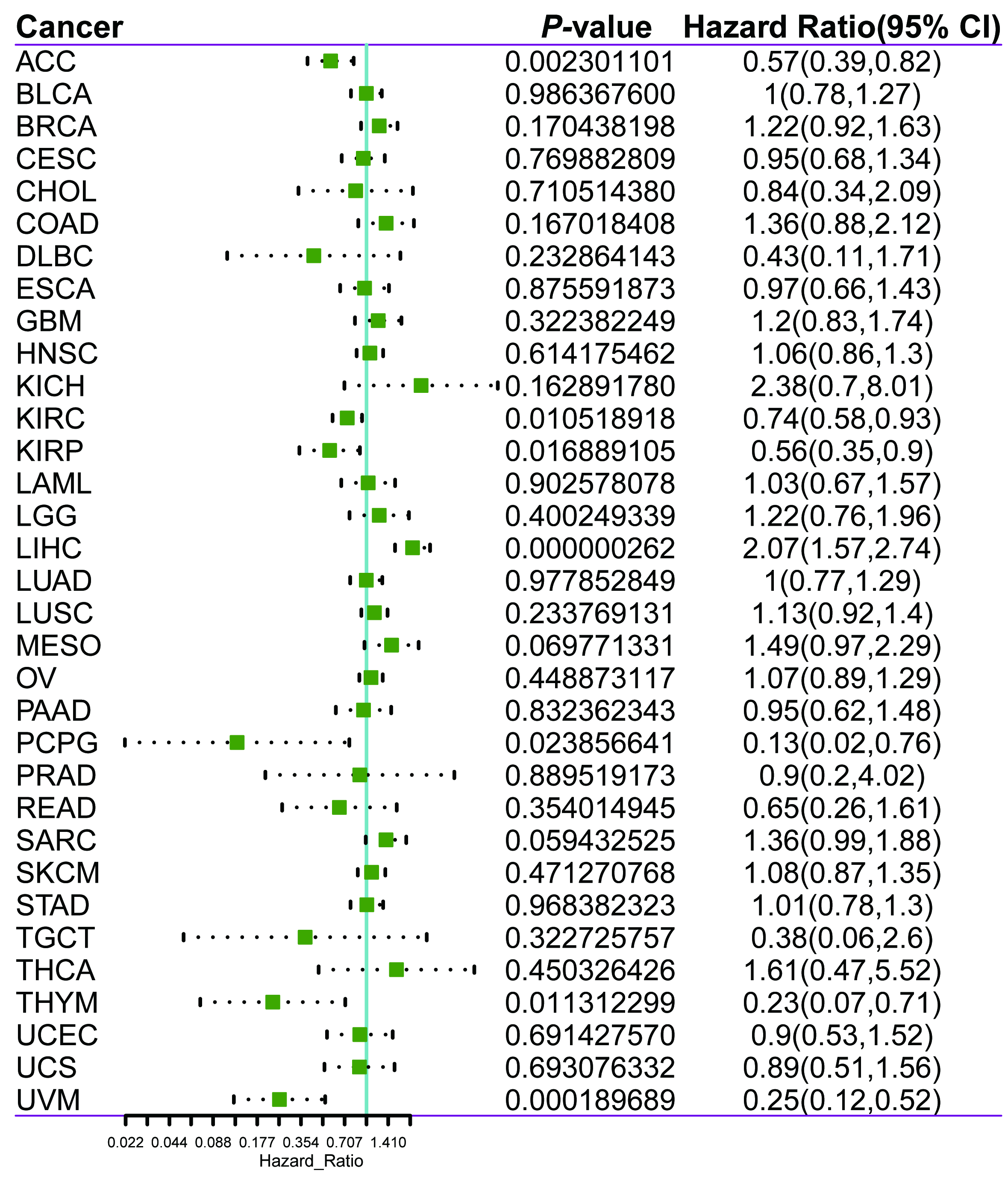

Supplement: Supplementary Figure 4 — Relationship between expression of SLC41A3 and overall survival in pan-cancer. Cox regression was used to examine the correlation, p< 0.05 was considered significant. [file Image_4.tif]

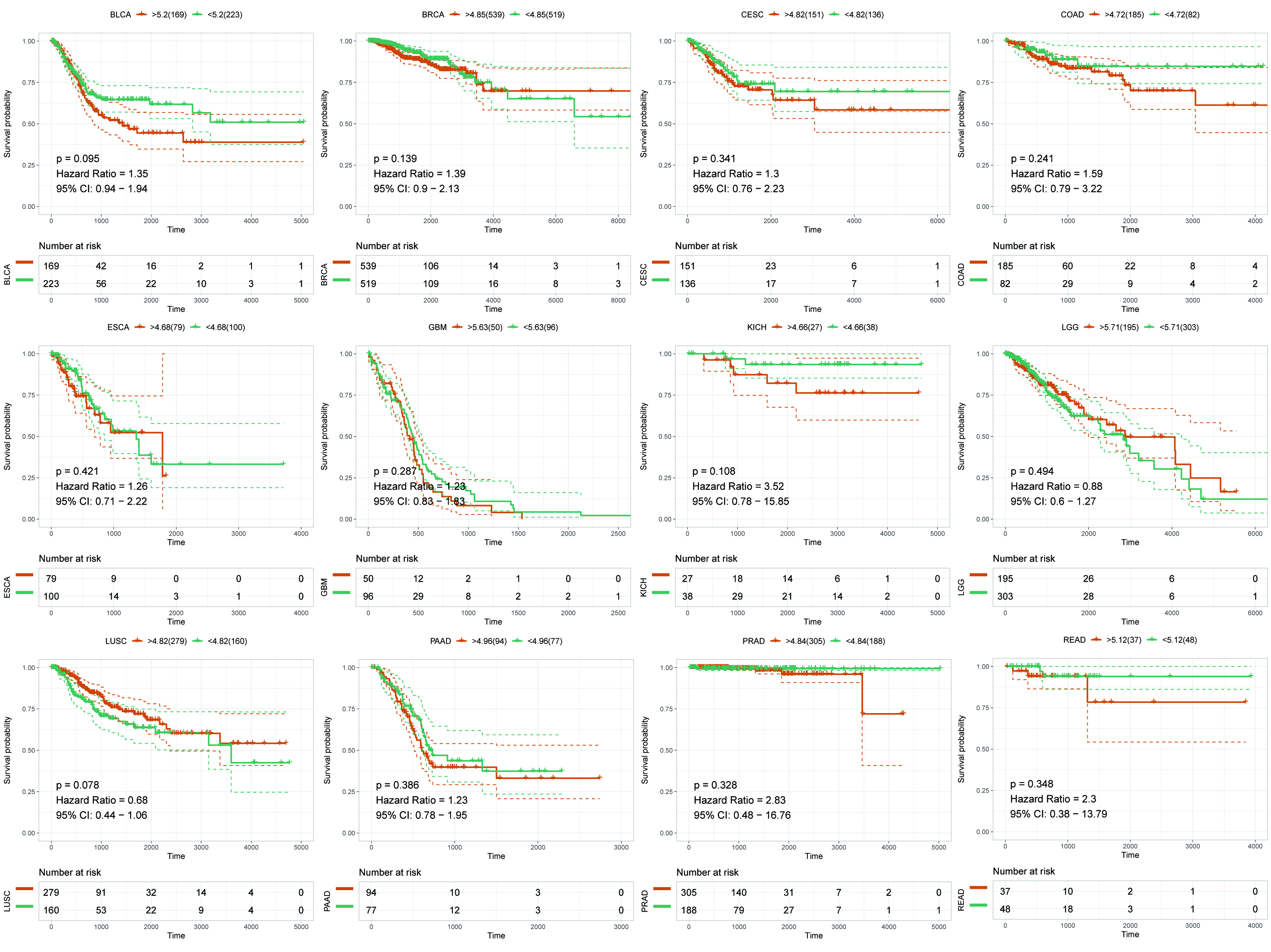

Supplement: Supplementary Figure 5 — Disease-specific Survival (DSS) of SLC41A3 in different cancer types. p< 0.05 was considered significant. [file Image_5.tif]

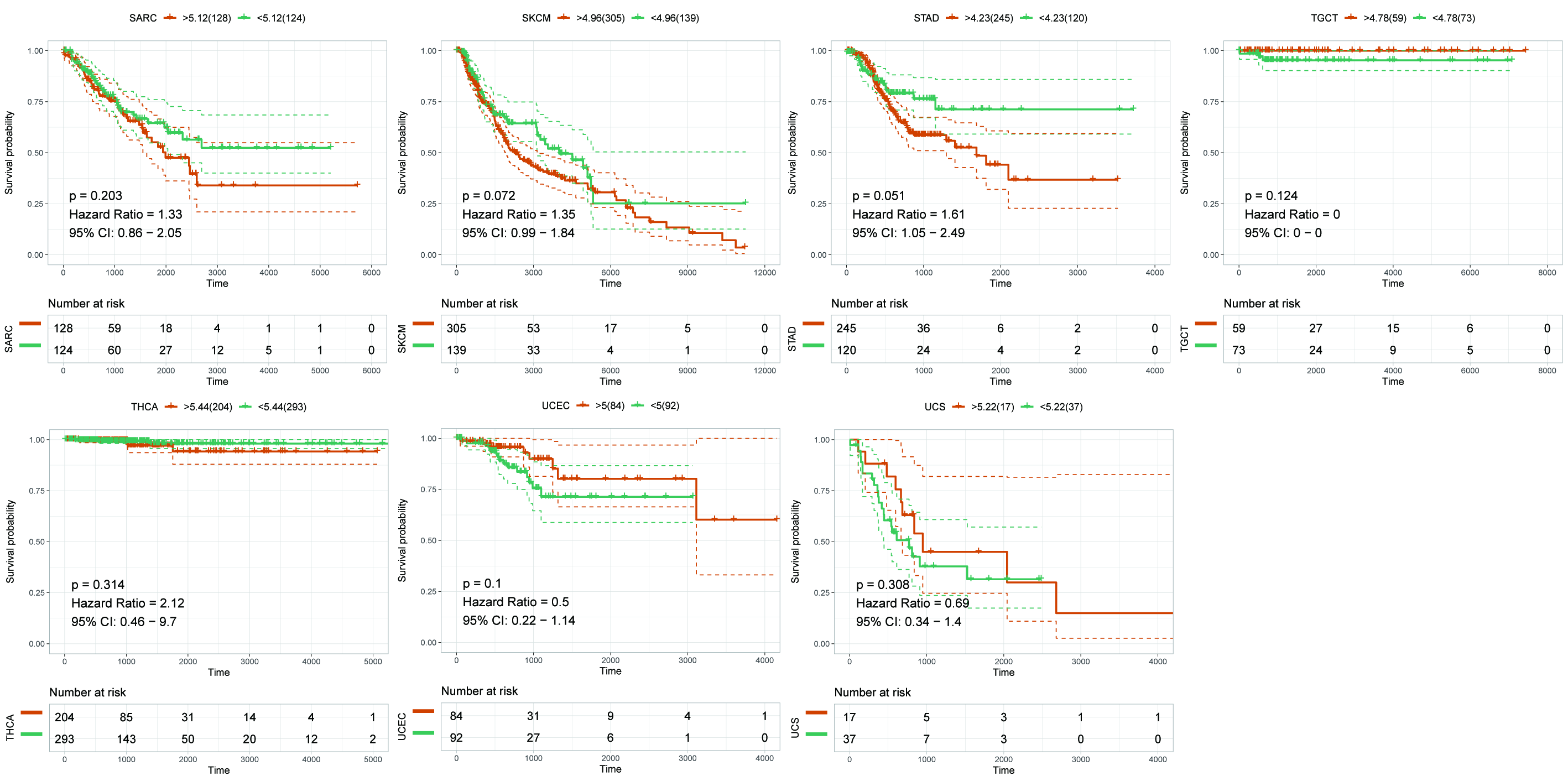

Supplement: Supplementary Figure 6 — Disease-specific Survival (DSS) of SLC41A3 in different cancer types. p< 0.05 was considered significant. [file Image_6.tif]

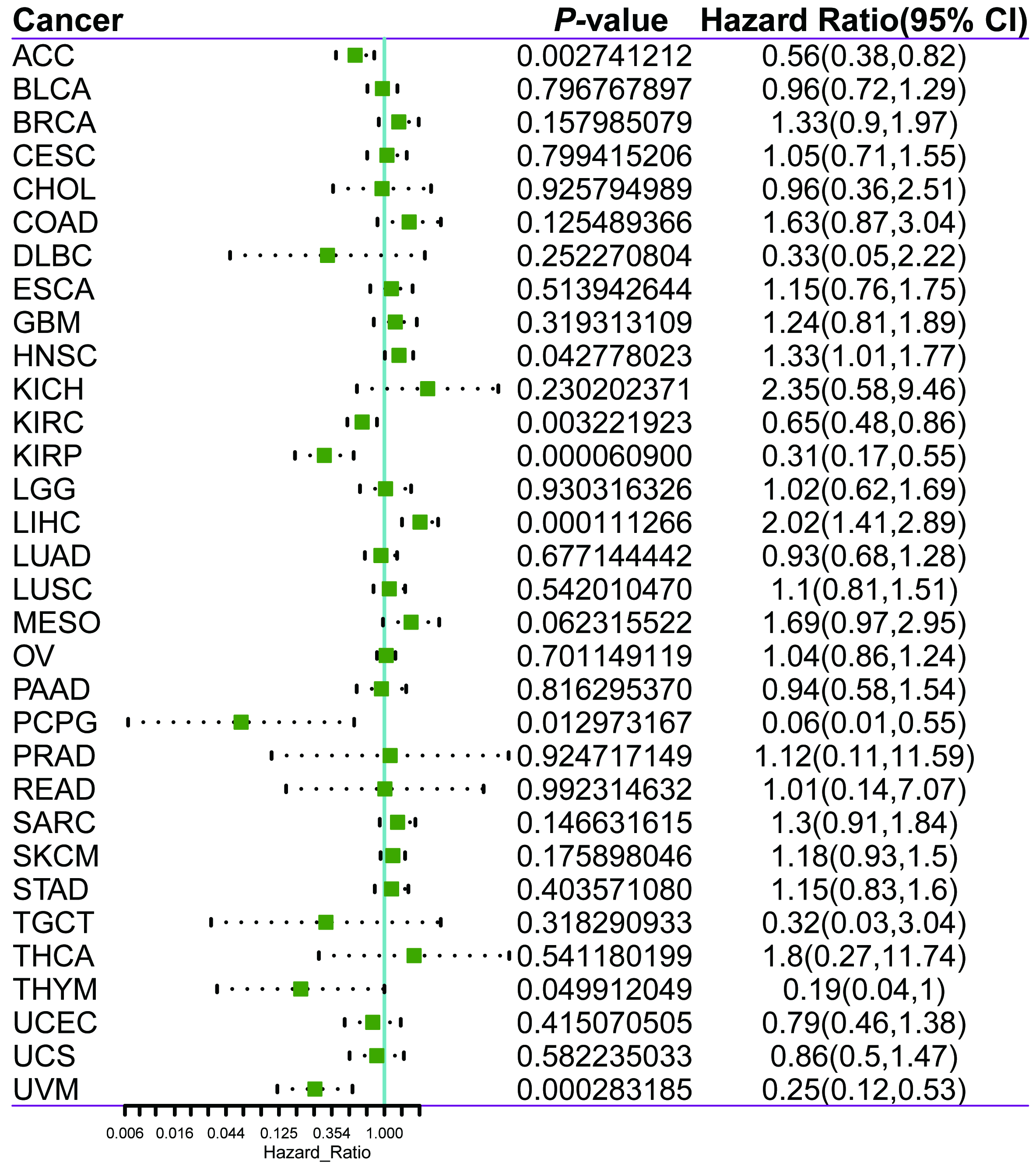

Supplement: Supplementary Figure 7 — Relationship between expression of SLC41A3 and disease-specific survival in pan-cancer. Cox regression was used to examine the correlation, p< 0.05 was considered significant. [file Image_7.tif]

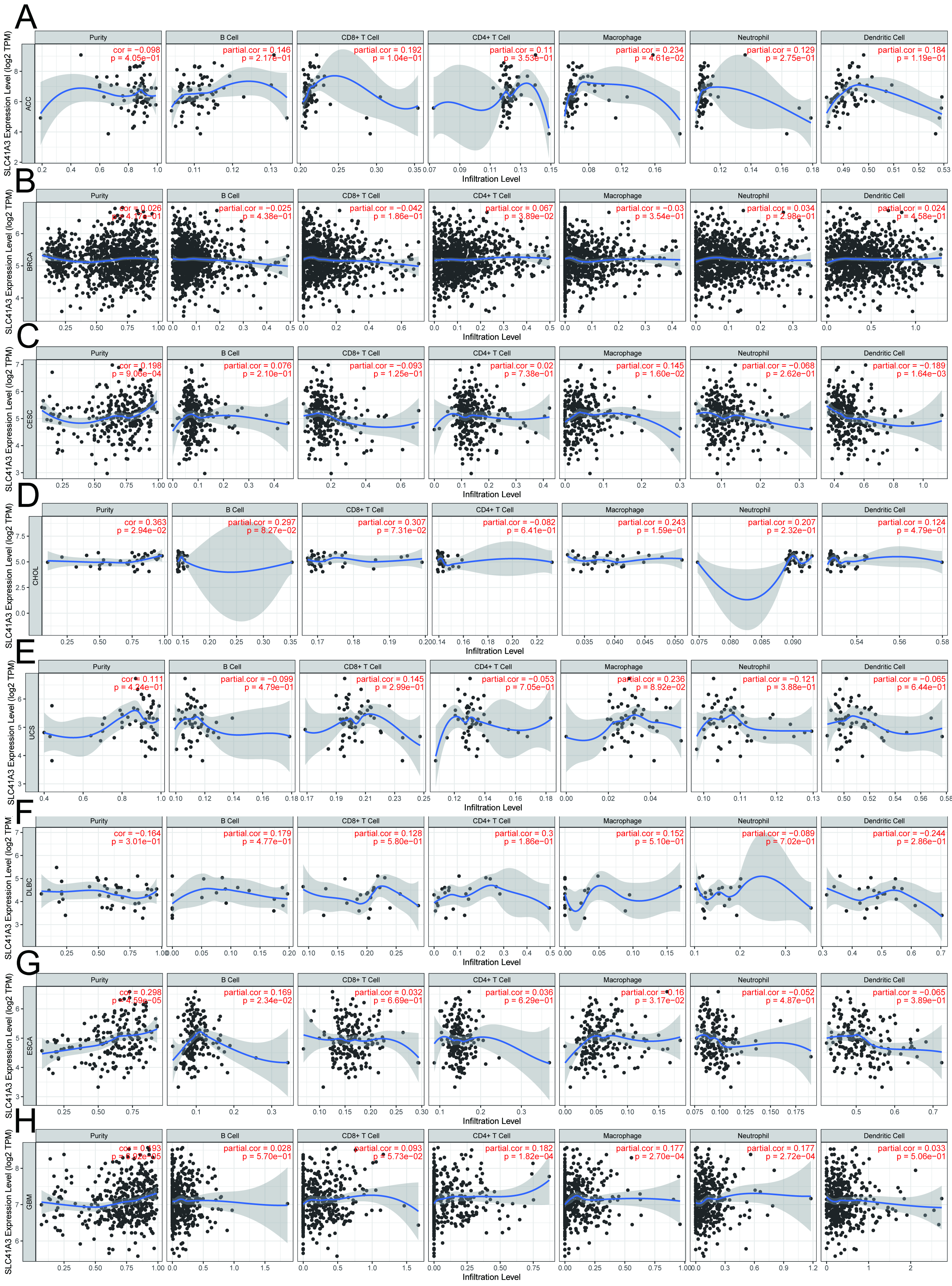

Supplement: Supplementary Figure 8 — Correlation between SLC41A3 expression level and immune cell infiltration. Correlation between six immune cell infiltration scores (B cell, CD4+ T cell, CD8+ T cell, Neutrophil, Macrophage, Dendritic cell) and SLC41A3 mRNA expression in (A) ACC, (B) BRCA, (C) CESC, (D) (CHOL), (E) UCS, (F) DLBC, (G) ESCA, (H) GBM. [file Image_8.tif]

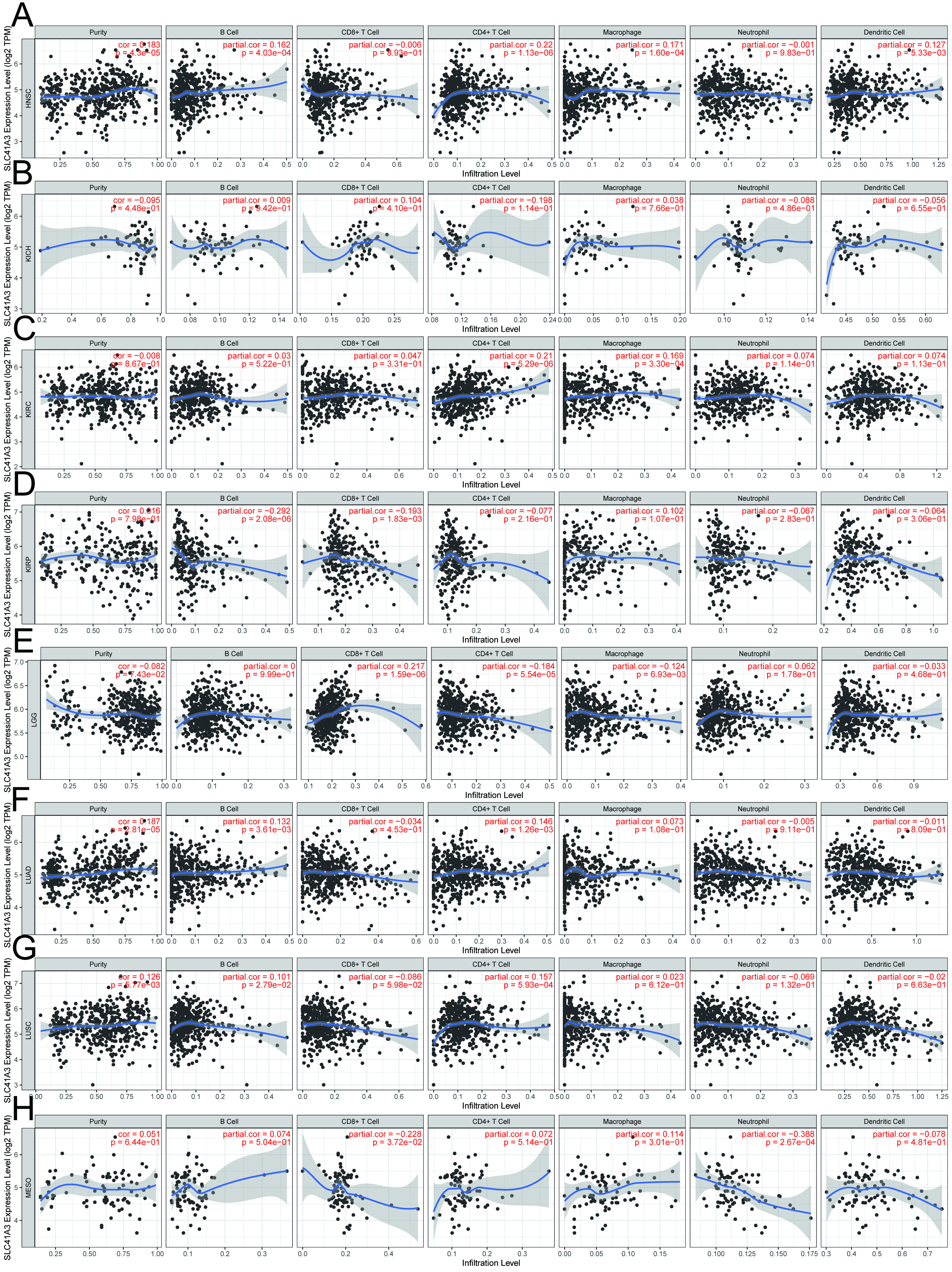

Supplement: Supplementary Figure 9 — Correlation between SLC41A3 expression level and immune cell infiltration. Correlation between six immune cell infiltration scores (B cell, CD4+ T cell, CD8+ T cell, Neutrophil, Macrophage, Dendritic cell) and SLC41A3 mRNA expression in (A) HNSC, (B) KICH, (C) KIRC, (D) KIRP, (E) LGG, (F) LUAD, (G) LUSC, (H) MESO. [file Image_9.tif]

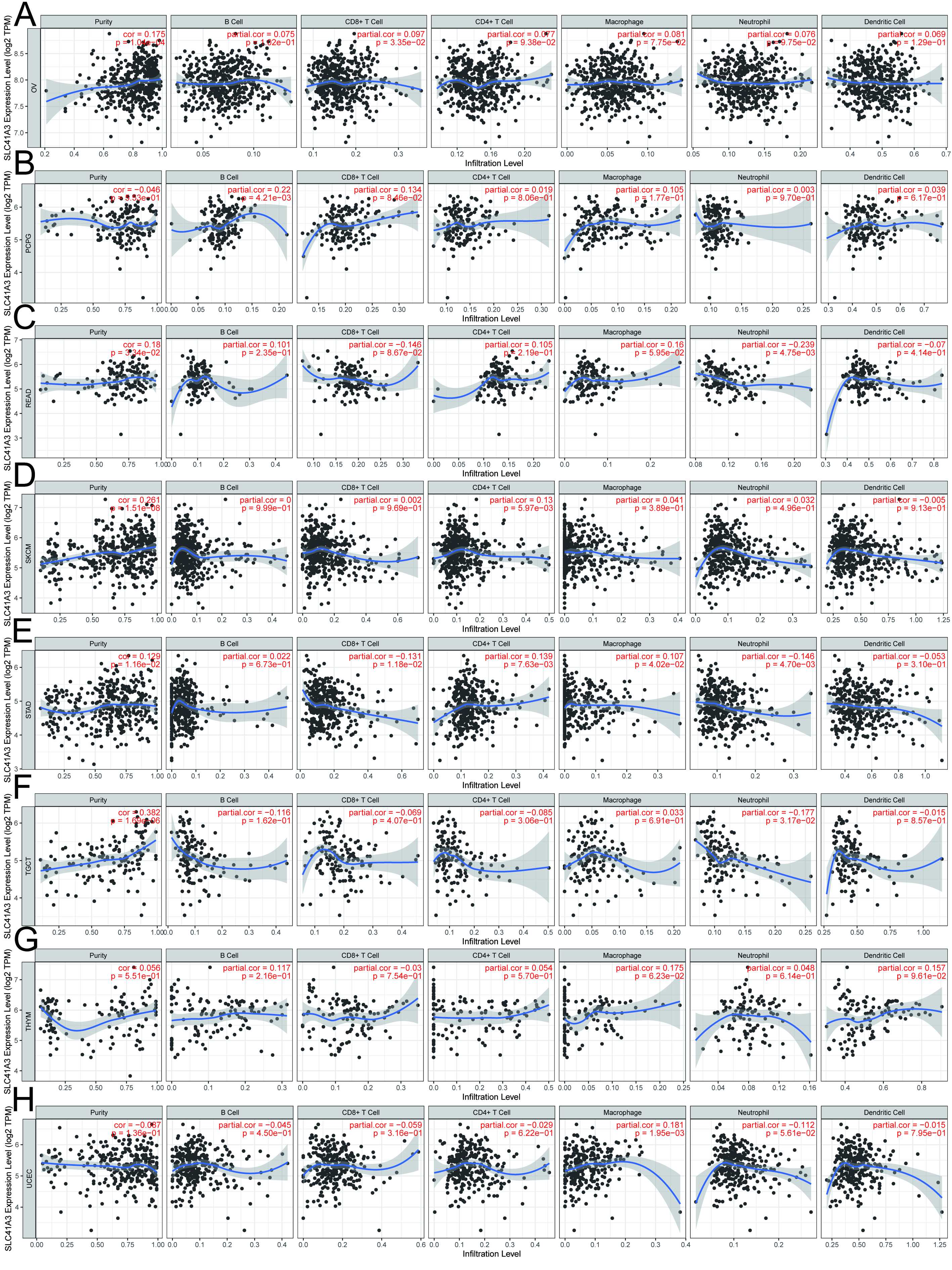

Supplement: Supplementary Figure 10 — Correlation between SLC41A3 expression level and immune cell infiltration. Correlation between six immune cell infiltration scores (B cell, CD4+ T cell, CD8+ T cell, Neutrophil, Macrophage, Dendritic cell) and SLC41A3 mRNA expression in (A) OV, (B) PCPG, (C) READ, (D) SKCM, (E) STAD, (F) TGCCT, (G) THYM, (H) UCEC. [file Image_10.tif]

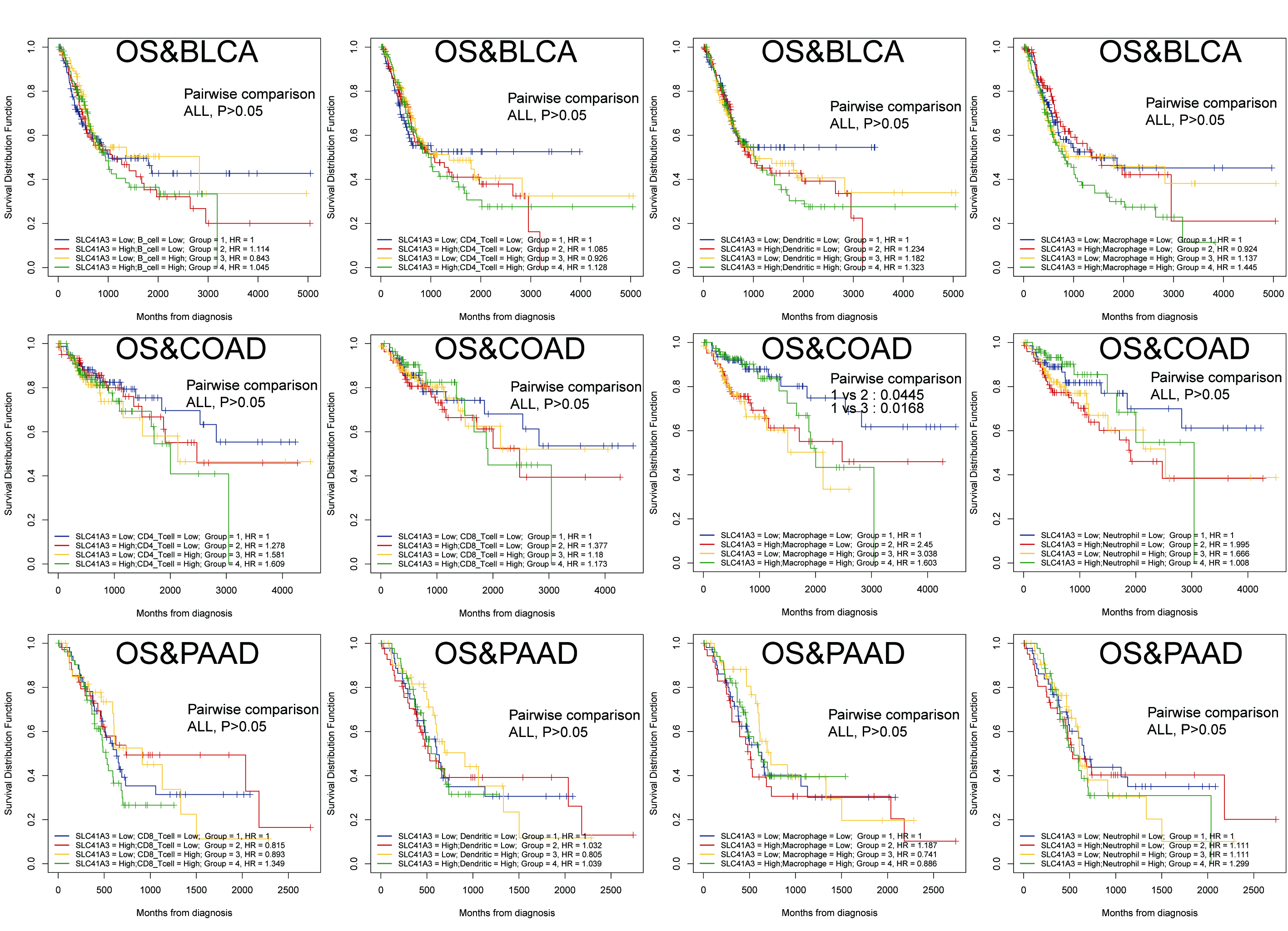

Supplement: Supplementary Figure 11 — Overall Survival (OS) curves using combinations SLC41A3 expression and Immune cells score. p< 0.05 was considered significant. [file Image_11.tif]

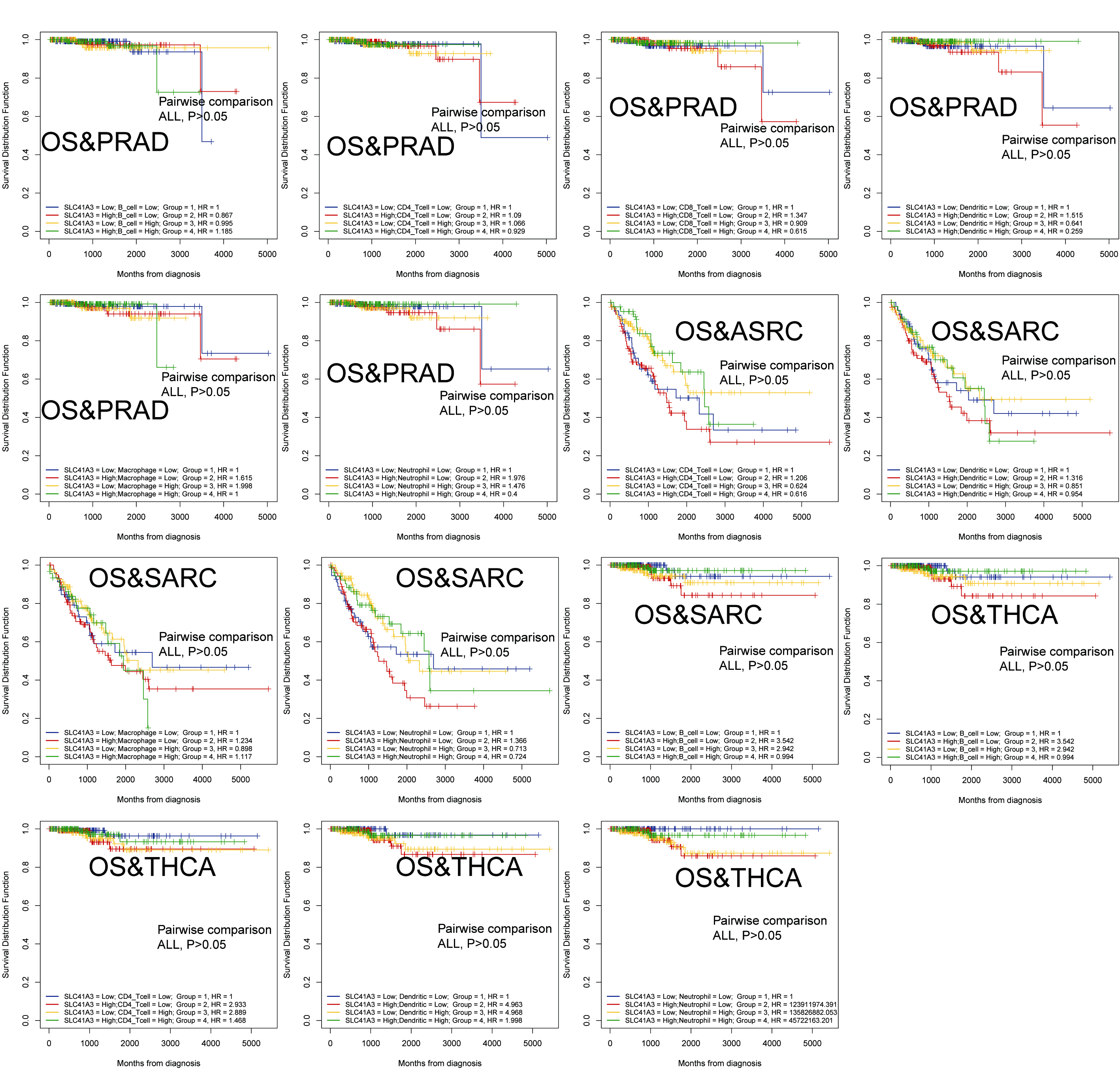

Supplement: Supplementary Figure 12 — Overall Survival (OS) curves using combinations SLC41A3 expression and Immune cells score. p< 0.05 was considered significant. [file Image_12.tif]

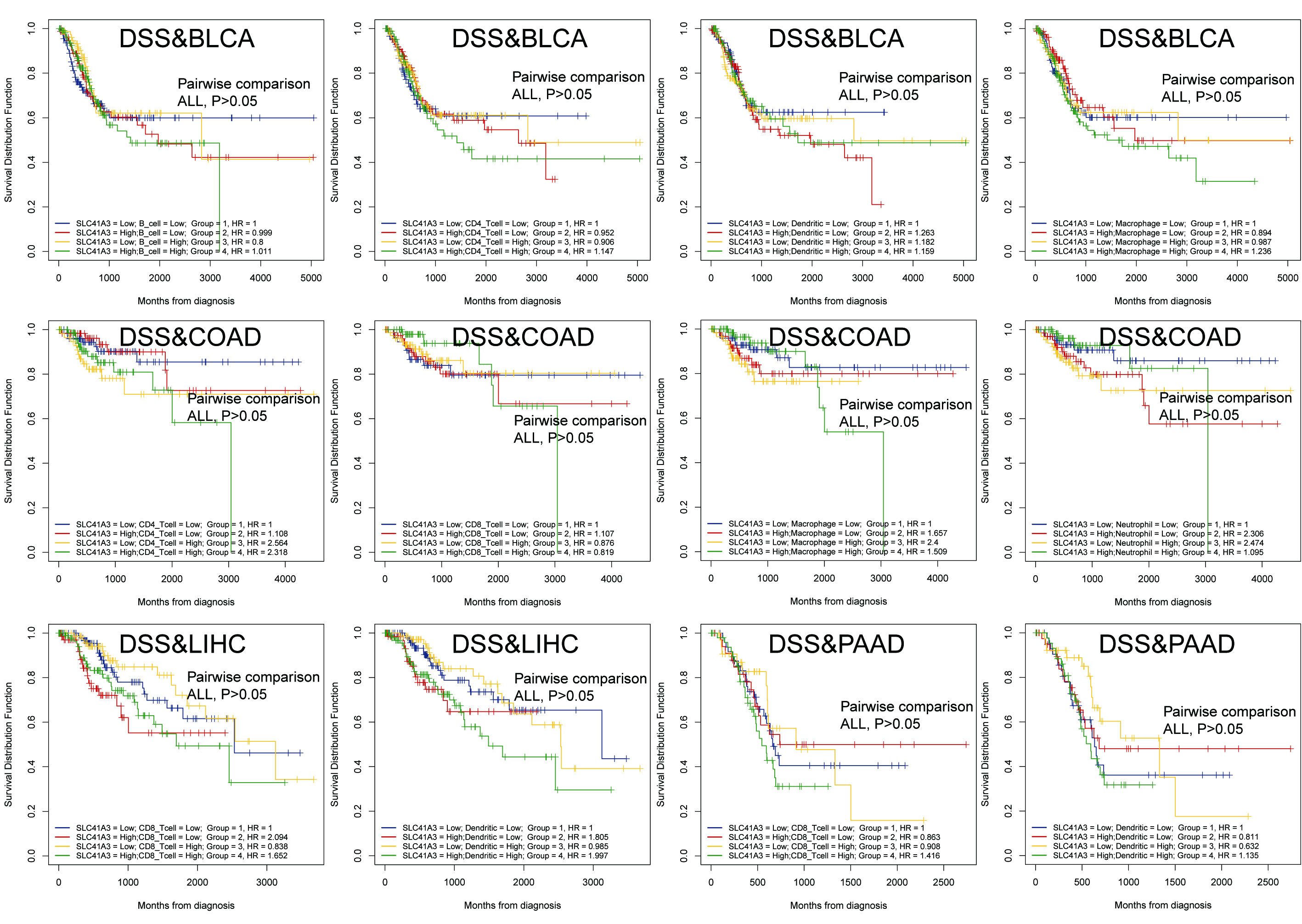

Supplement: Supplementary Figure 13 — Disease-specific Survival (DSS) curves using combinations SLC41A3 expression and Immune cells score. p< 0.05 was considered significant. [file Image_13.tif]

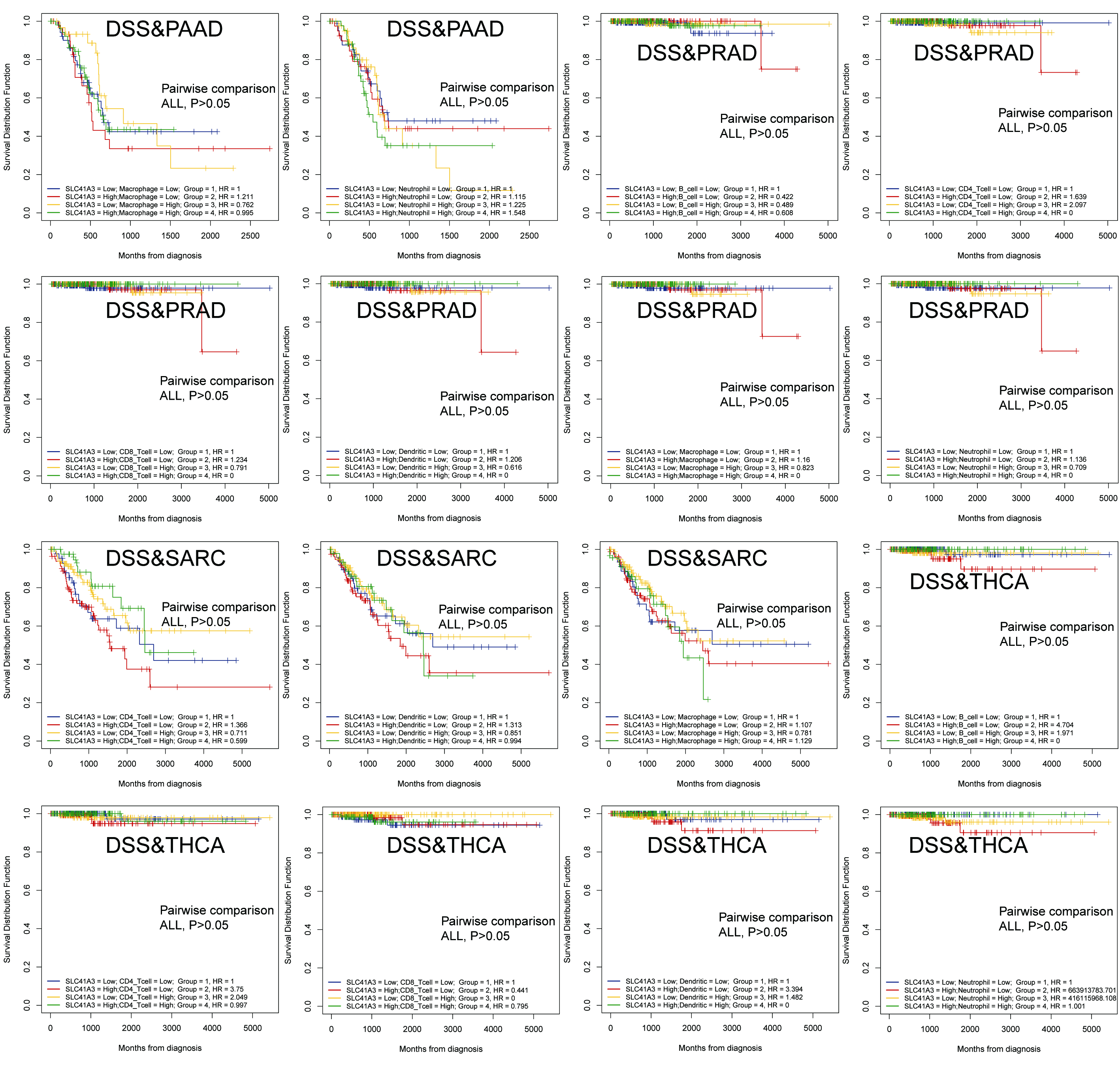

Supplement: Supplementary Figure 14 — Disease-specific Survival (DSS) curves using combinations SLC41A3 expression and Immune cells score. p< 0.05 was considered significant. [file Image_14.tif]

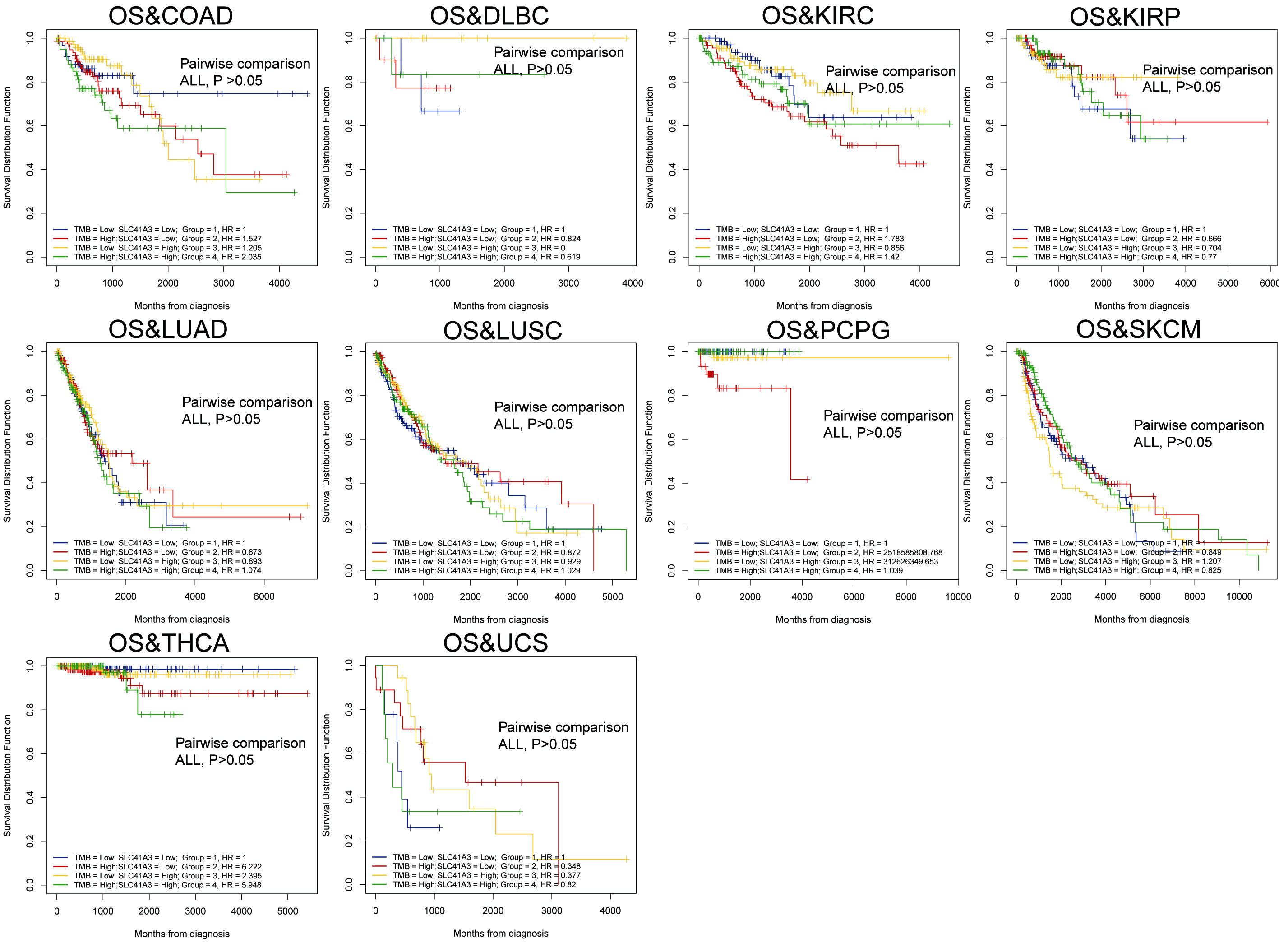

Supplement: Supplementary Figure 15 — Overall survival (OS) analysis combinations SLC41A3 expression and tumor mutation burden (TMB). p< 0.05 was considered significant. [file Image_15.tif]

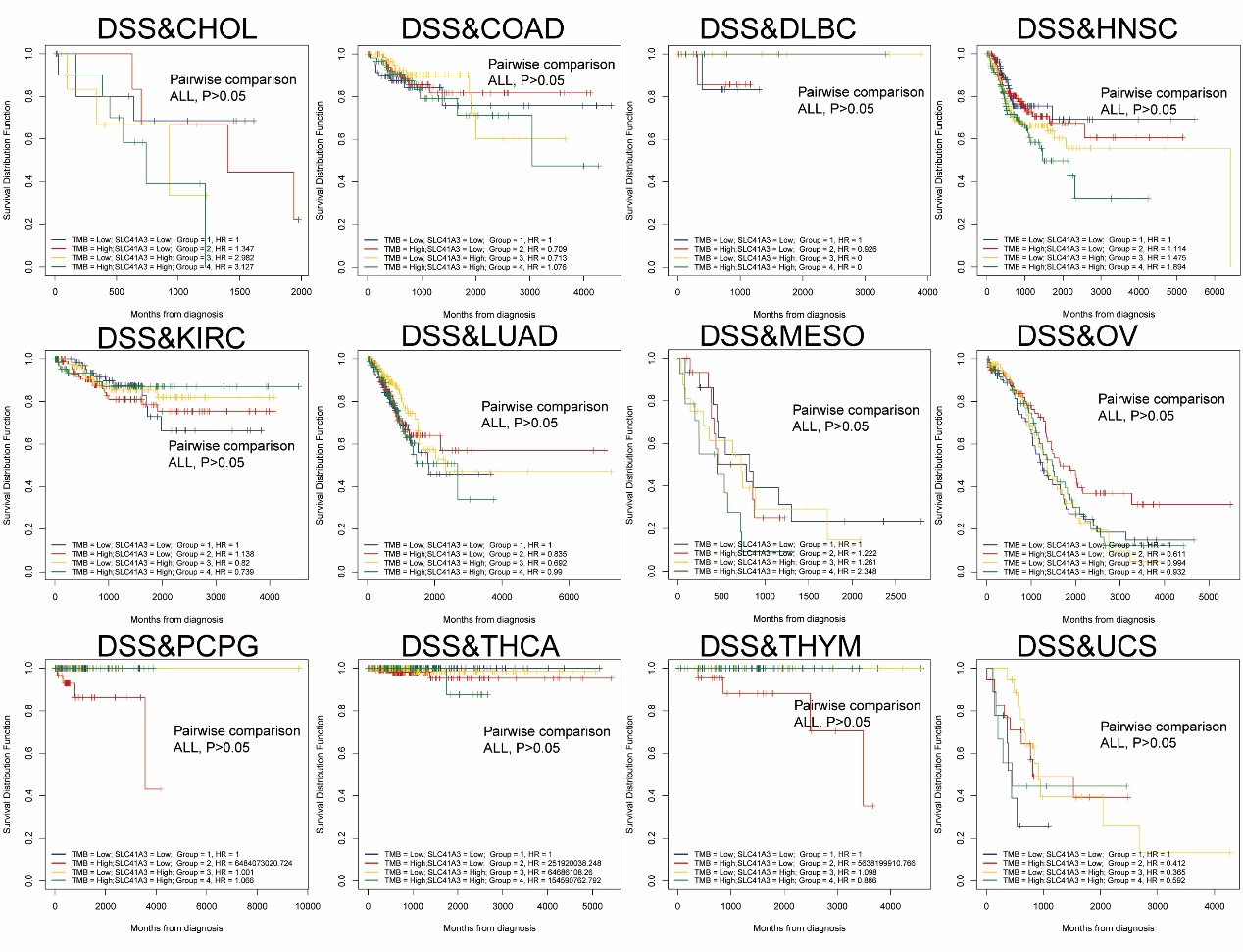


FIG S16.


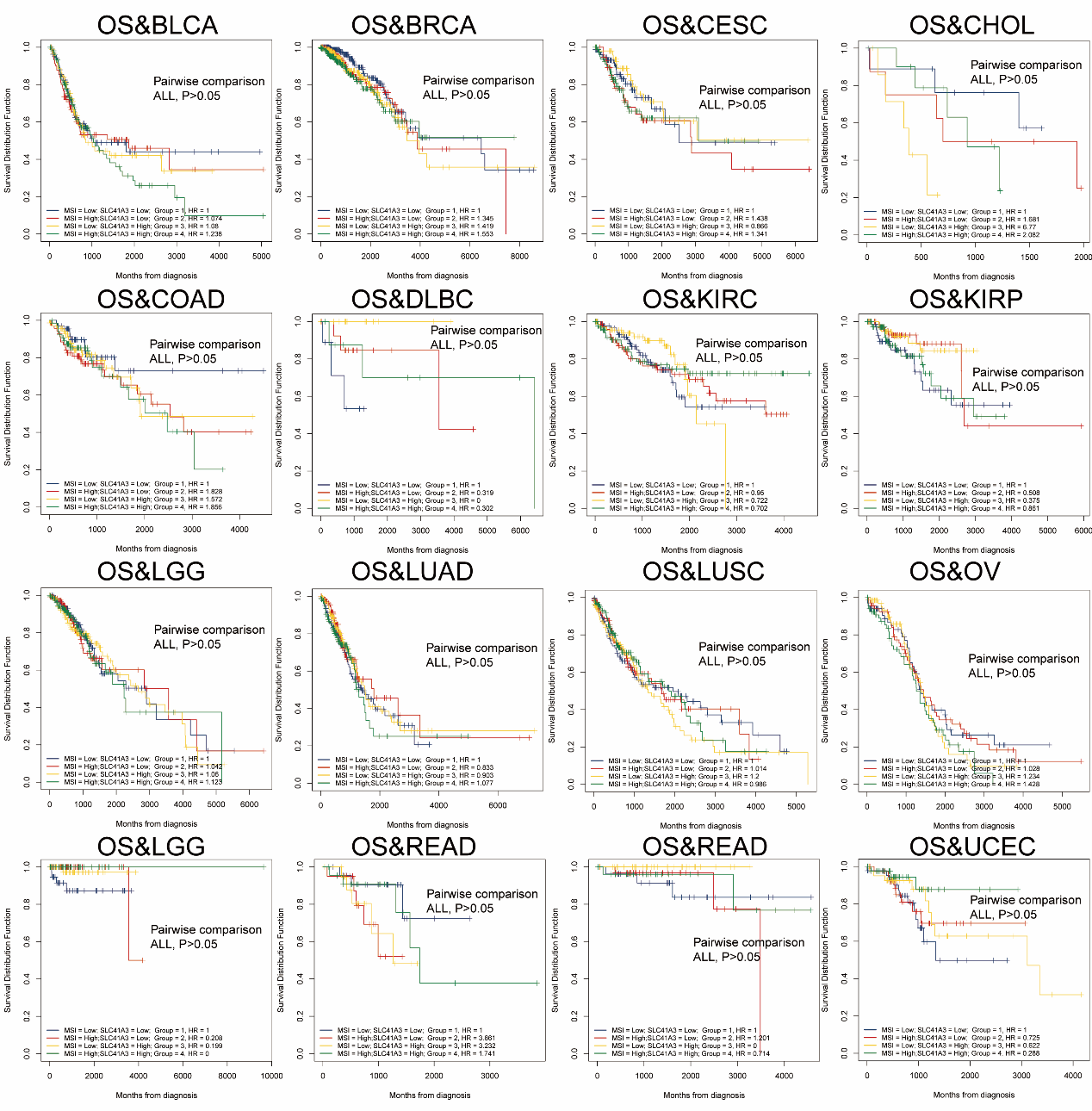


FIG S17


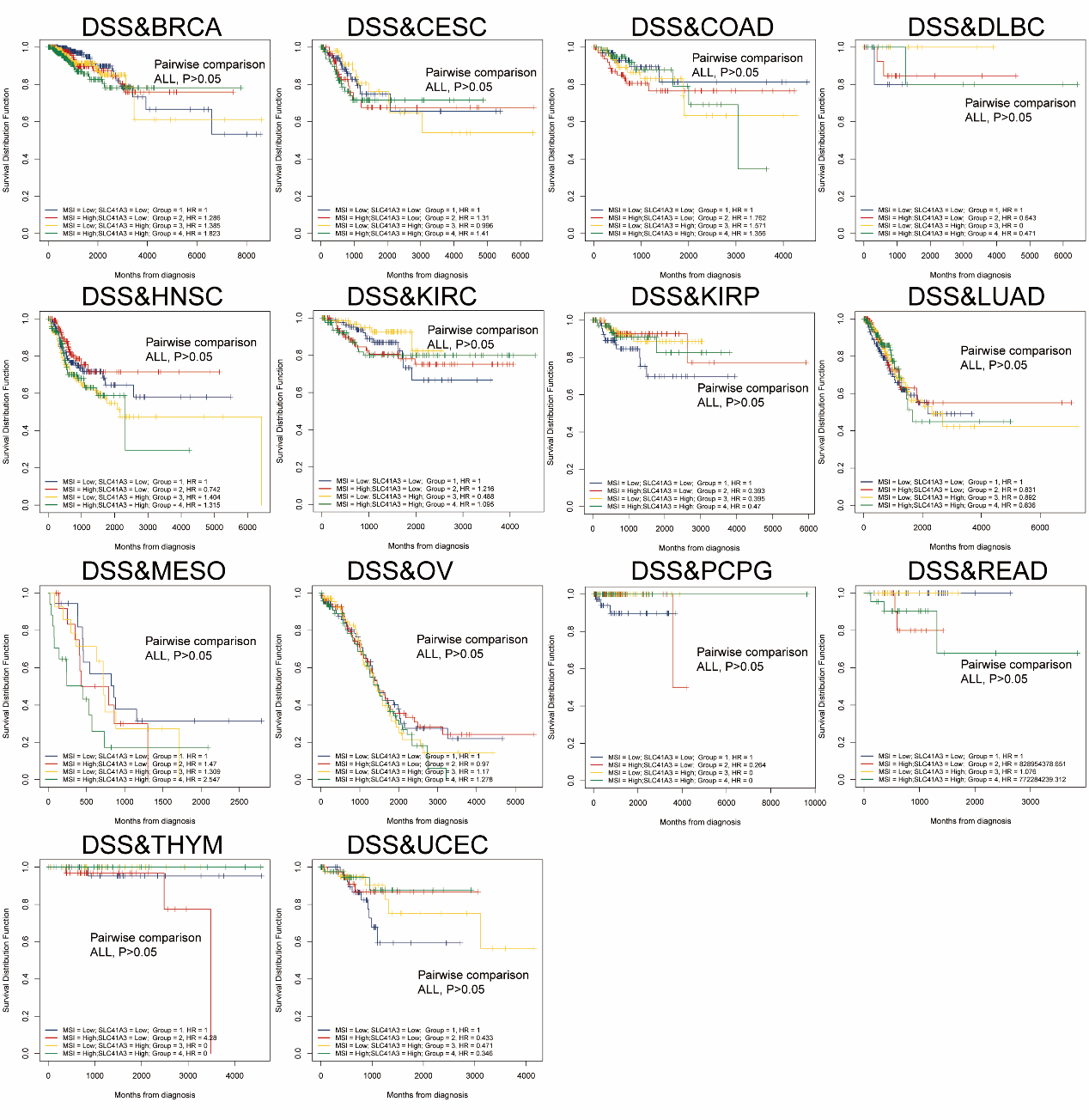


FIG S18


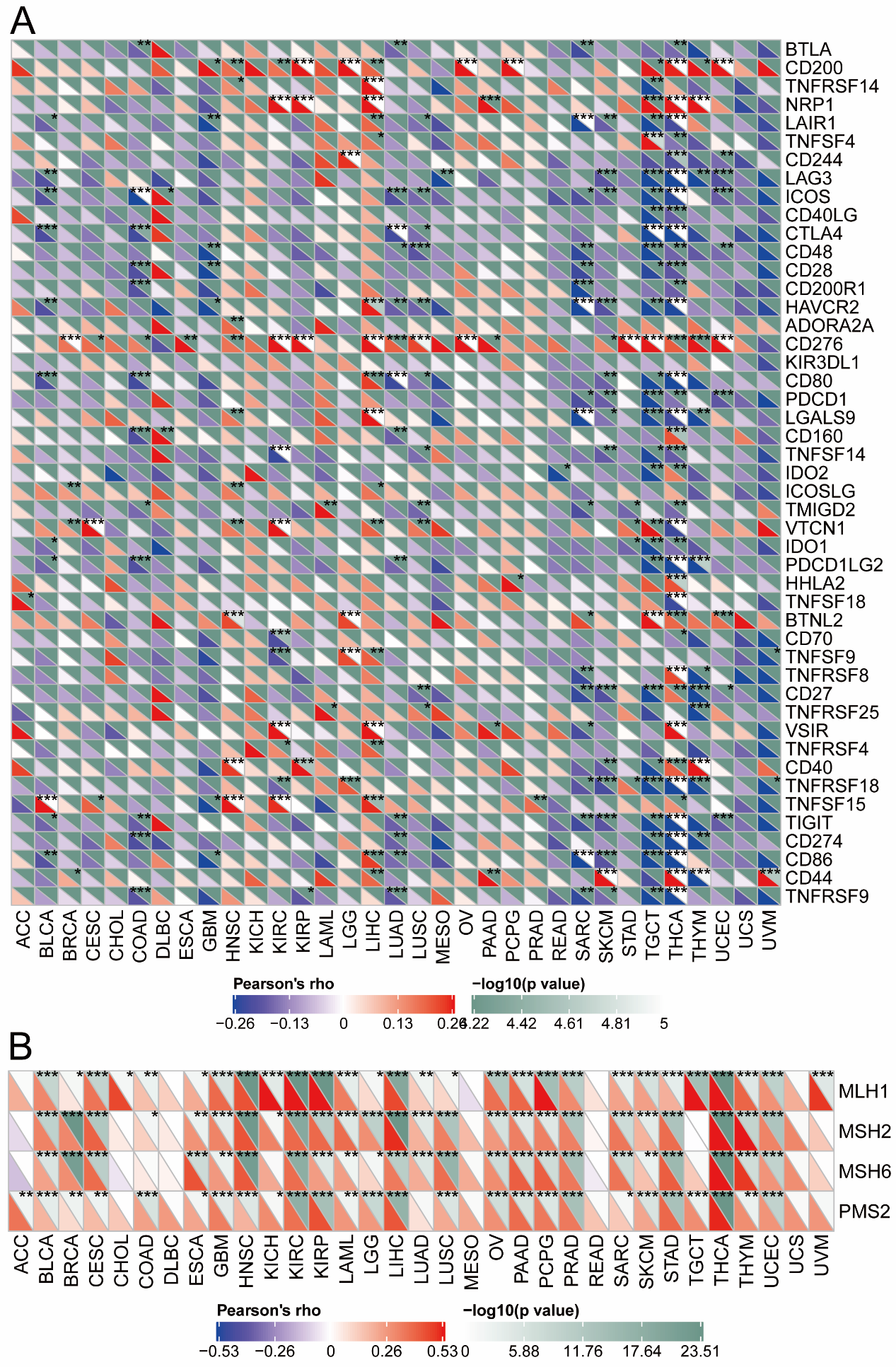


FIG S19


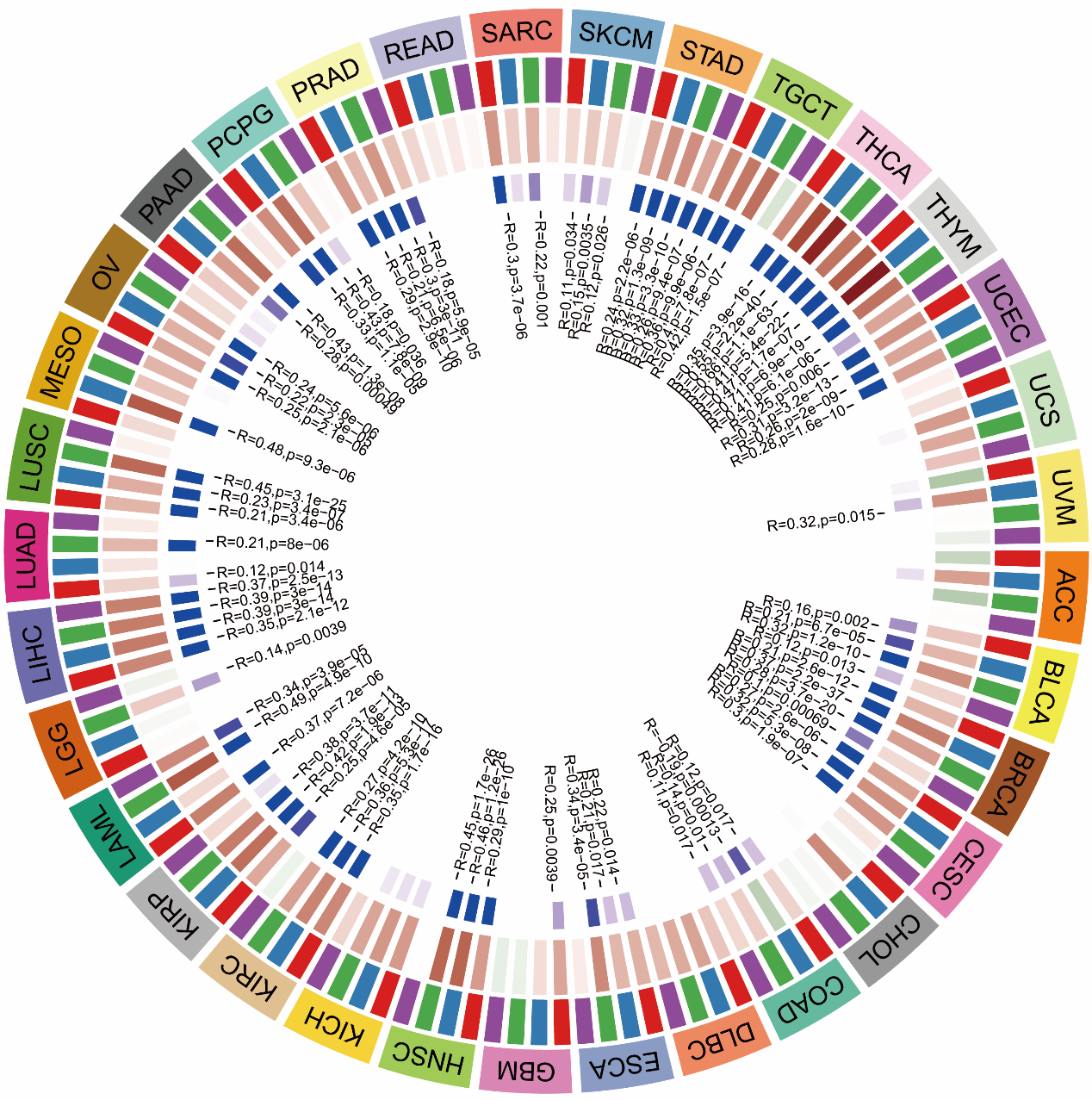


FIG S20


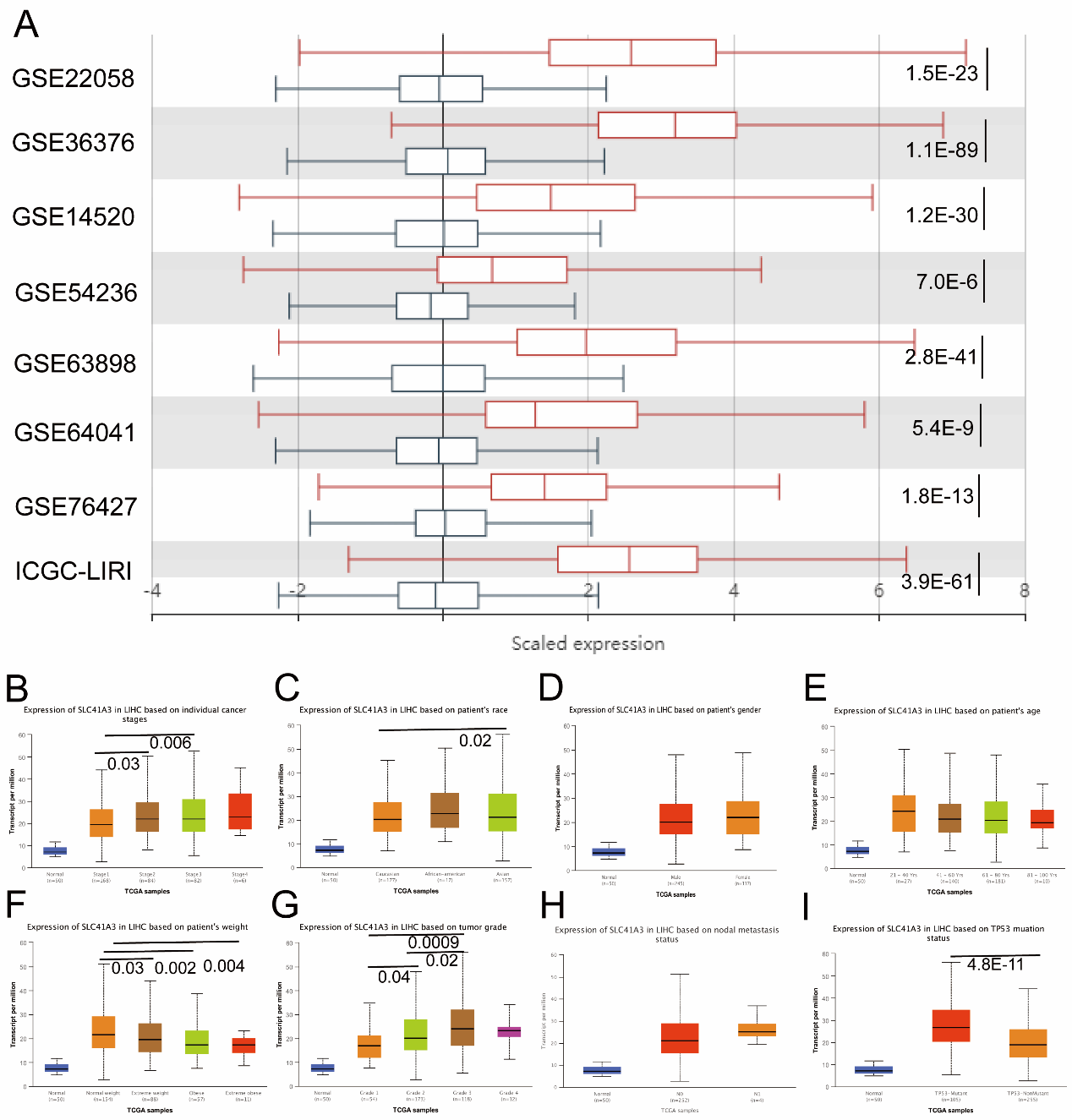


FIG S21


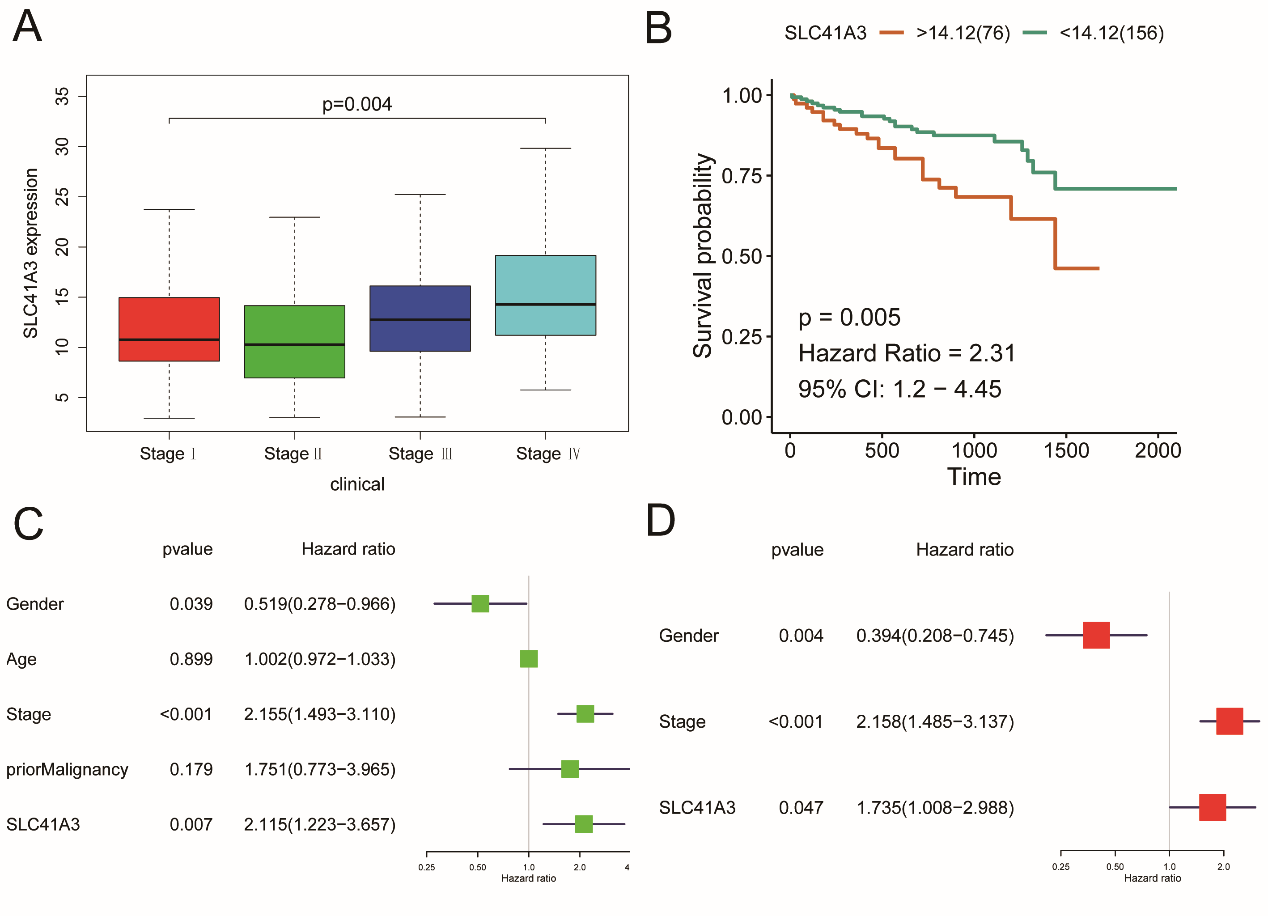


FIG S22


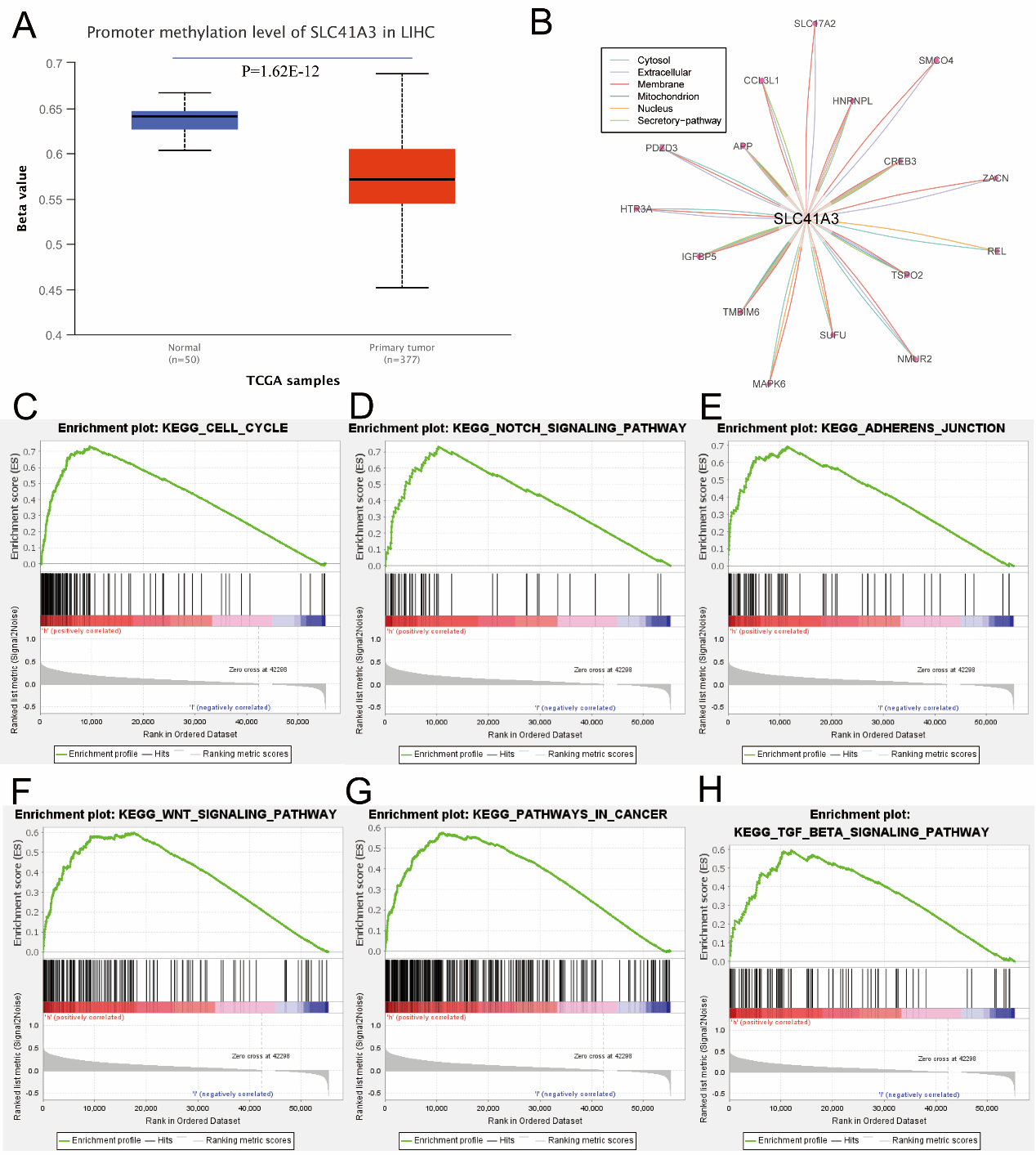


FIG S23

Supplement: Supplementary Figure 16 — Disease-specific Survival (DSS) analysis combinations SLC41A3 expression and tumor mutation burden (TMB). p< 0.05 was considered significant. [file DataSheet_3.docx]
